# Heteroatom-Doped Carbon Quantum Dots and Polymer Composite as Dual-Mode Nanoprobe for Fluorometric and Colorimetric Determination of Picric Acid

**DOI:** 10.1021/acsami.3c07938

**Published:** 2023-08-23

**Authors:** Ömer Kaan Koç, Ayşem Üzer, Reşat Apak

**Affiliations:** † Institute of Graduate Studies, Istanbul University-Cerrahpaşa, Avcilar, Istanbul 34320, Turkey; ‡ Department of Chemistry, Faculty of Engineering, Istanbul University-Cerrahpaşa, Avcilar, Istanbul 34320, Turkey; § Bayraktar Neighborhood, Turkish Academy of Sciences (TUBA), Vedat Dalokay Street No: 112, Çankaya, Ankara 06690, Turkey

**Keywords:** carbon quantum dots, dual-mode nanosensor, picric acid detection, inner filter effect, fluorescence quenching, PVA-based polymer film

## Abstract

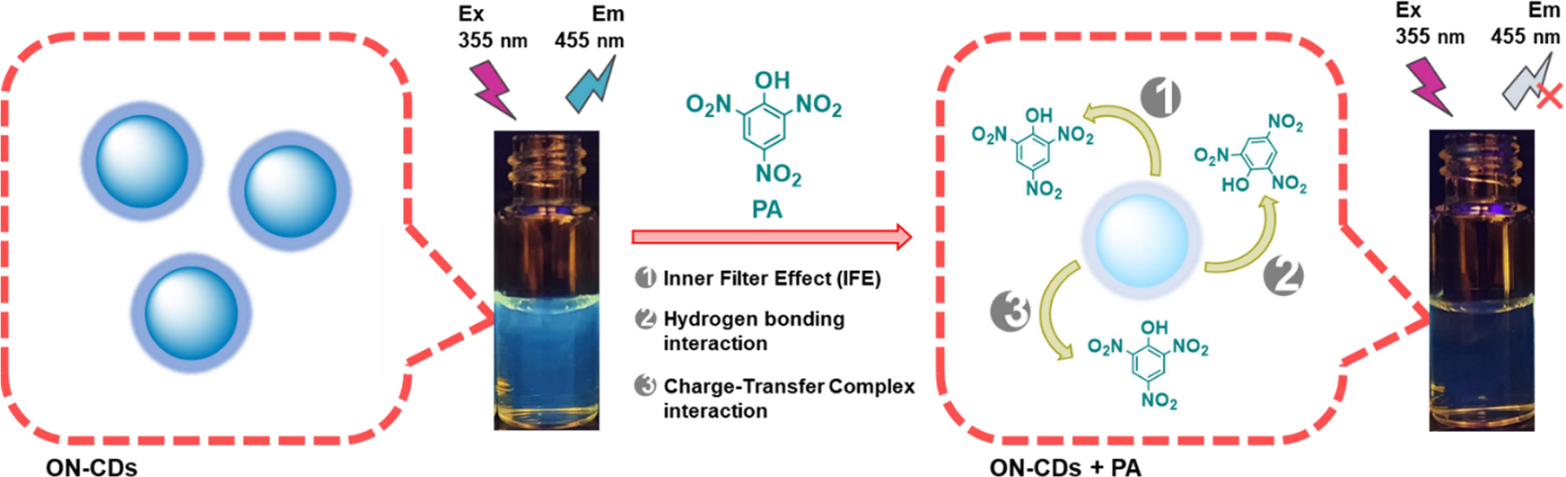

Oxygen- and nitrogen-heteroatom-doped, water-dispersible, and bright blue-fluorescent carbon dots (ON–CDs) were prepared for the selective and sensitive determination of 2,4,6-trinitrophenol (picric acid, PA). ON–CDs with 49.7% quantum yield were one-pot manufactured by the reflux method using citric acid, d-glucose, and ethylenediamine precursors. The surface morphology of ON–CDs was determined by scanning transmission electron microscopy, high-resolution transmission electron microscopy, dynamic light scattering, Raman, infrared, and X-ray photoelectron spectroscopy techniques, and their photophysical properties were estimated by fluorescence spectroscopy, UV–vis spectroscopy, fluorescence lifetime measurement, and 3D-fluorescence excitation–emission matrix analysis. ON–CDs at an average particle size of 3.0 nm had excitation/emission wavelengths of 355 and 455 nm, respectively. With the dominant inner-filter effect- and hydrogen-bonding interaction-based static fluorescence quenching phenomena supported by ground-state charge-transfer complexation (CTC), the fluorescence of ON–CDs was selectively quenched with PA in the presence of various types of explosives (i.e., 2,4,6-trinitrotoluene, tetryl, 1,3,5-trinitroperhydro–1,3,5-triazine, 1,3,5,7-tetranitro-1,3,5,7-tetraazacyclooctane, pentaerythritol tetranitrate, 3-nitro-1,2,4-triazole-5-one, and TATP-hydrolyzed H_2_O_2_). The analytical results showed that the emission intensity varied linearly with a correlation coefficient of 0.9987 over a PA concentration range from 1.0 × 10^–9^ to 11.0 × 10^–9^ M. As a result of ground-state interaction (H-bonding and CTC) of ON–CDs with PA, an orange-colored complex was formed different from the characteristic yellow color of PA in an aqueous medium, allowing naked-eye detection of PA. The detection limits for PA with ON–CDs were 12.5 × 10^–12^ M (12.5 pM) by emission measurement and 9.0 × 10^–10^ M (0.9 nM) by absorption measurement. In the presence of synthetic explosive mixtures, common soil cations/anions, and camouflage materials, PA was recovered in the range of 95.2 and 102.5%. The developed method was statistically validated against a reference liquid chromatography coupled to tandem mass spectrometry method applied to PA-contaminated soil. In addition, a poly(vinyl alcohol)-based polymer composite film {PF(ON–CDs)} was prepared by incorporating ON–CDs, enabling the smartphone-assisted fluorometric detection of PA.

## Introduction

2,4,6-trinitrophenol (TNP), with the widely used name, picric acid (PA), is an easily accessible and dangerous nitroaromatic with strong electron-withdrawing groups.^1^ Despite attracting less attention than its trinitro analog, 2,4,6-trinitrotoluene (TNT), PA actually has a more potent explosive effect than TNT.^2^ Picric acid and picrates as PA salts are the main components of explosive formulations such as pentolite, yellow-D, and explosive-D. Due to the relative sensitivity of PA and picrates to shock and friction, their use in ammunition declined after World War-I. PA and picrates are also used in armor-piercing projectiles, bombs, rocket warheads, grenades, and minelayers.^3,4^ Apart from the production of explosives, PA is widely used as a raw material in aniline synthesis, leather, paint, glass, and fireworks industries.^5,6^ As a result of its diverse usage, PA can pass to the environment while being produced, transported, used, and disposed of, thereby polluting the soil and groundwater.^7^ Therefore, there is a need for developing new methods to detect PA sensitively and selectively in aqueous media and soil.

PA can be enriched by liquid–liquid microextraction techniques;^8^ current methods developed for PA determination include surface plasmon resonance-based immunosensing,^9^ and proton transfer-assisted soft chemical ionization mass spectrometry.^10^ It can be argued that these existing methods restrict practical applicability, are expensive, and involve laborious procedures that may pose difficulties in field use.^11^ In comparison with conventional detection methods, nanoparticle-, polymeric nanocomposite-, and MOF-based sensing elements have been increasingly designed in recent years because these rapid-response sensors are available, portable, and easily applicable to diverse media.^12−14^ Especially carbon dots, being one of the most widely exploited groups in nanoscience, display an ascending trend.^15^ As they are easily synthesized with high quantum yield (QY) and low cost, are photostable, and have high surface area and fluorescence, they naturally attract the attention of scientists and technologists.

Carbon dots are recently developed as extraordinarily bright fluorescent carbon nanoparticles.^16^ They display many excellent environmentally friendly properties, such as easy synthesis, sufficient water solubility and small size, finely tuned excitation/emission spectra, good biocompatibility, and photostability.^17,18^ Carbon dots are diversely applied in bioimaging,^19^ medical diagnostics,^20^ catalysis,^21^ photovoltaic devices,^22^ and chemical sensors.^23^ Surface passivation as a result of heteroatom doping with N, O, P, and S constitutes a smart and powerful approach to promote the fluorescence of carbon dots.^24^ In particular, this technology is actively used to tune the emission band and to achieve a high quantum efficiency for carbon dots with nitrogen-doping or codoping with nitrogen and another element (such as O, S, and P).^25^

Here, carbon dots doped with oxygen and nitrogen (named ON–CDs) were manufactured by the reflux method using citric acid (CA), d-glucose (Glu), and ethylenediamine (EDA) precursors for sensitive and selective determination of PA. ON–CDs at an elevated high QY of 49.7% and an average particle size of 3.0 nm were able to detect PA in aqueous medium with high sensitivity and selectivity. ON–CDs, forming an orange complex with PA in the ground state, enabled PA determination with the naked eye. PA could be determined with high sensitivity in mixtures prepared with different explosives or their hydrolyzates [i.e., TNT, tetryl, 1,3,5-trinitroperhydro–1,3,5-triazine (RDX), 1,3,5,7-tetranitro-1,3,5,7-tetraazacyclooctane (HMX), 3-nitro-1,2,4-triazole-5-one (NTO), pentaerythritol tetranitrate (PETN), and H_2_O_2_]. A number of metal cations, common anions, and camouflage materials used to disguise explosives that are likely to interfere did not significantly affect the developed method. Additionally, a poly(vinyl alcohol) (PVA)-based polymer film {PF(ON–CDs)} developed for the practical application of ON–CDs quenched fluorescence by interacting with PA at different concentrations, enabling easy PA estimation with smartphone support. The fluorometric method utilizing these ON–CDs could be easily used to determine PA in sandy soils.

## Experimental Section

### Materials

CA anhydrous, Glu, EDA, 2-[4-(2-hydroxyethyl)piperazine-1-yl]ethanesulfonic acid (HEPES), anthracene, PVA, acetone, ammonium acetate (NH_4_Ac), and hydrogen peroxide (H_2_O_2_) were purchased from Sigma (St. Louis, Missouri, USA). 2-amino-2-(hydroxymethyl)-1,3-propanediol (Tris), dimethyl sulfoxide, ethanol, methanol, and metal salts (CaCl_2_, ZnCl_2_, NaCl, MnCl_2_, FeCl_2_, CuCl_2_, MgCl_2_, CdCl_2_, NaNO_3_, NaNO_2_, Na_2_SO_4_, and Na_2_CO_3_) were obtained from Merck (Darmstadt, Germany). All chemicals used were of analytical reagent grade.

The explosive substances TNP (PA, pure), HMX (pure), RDX (which contains 85% active compound), TNT (pure), PETN (pure), tetryl (pure), NTO (pure), and composite B (containing RDX 60%, TNT 39%, and wax 1%) were kindly supplied by the Mechanical and Chemical Industry Corporation (Makine Kimya Endüstrisi Kurumu–MKEK; Ankara, Turkey) from previous projects. RTC blended clean sandy soil, used as a sample matrix for the determination of PA explosives, was obtained from Sigma–Aldrich (St. Louis, Missouri, USA).

All aqueous solutions used in the experiments were prepared with ultrapure water (18.2 MΩ·cm, Millipore).

### Apparatus

Fluorescence emission and excitation spectra were recorded by a Cary Eclipse fluorescence spectrophotometer (Agilent, USA) with Suprasil quartz cuvettes (Hellma Analytics, USA). Ultraviolet–visible absorption spectra were measured by a UV–1900i UV–vis spectrophotometer (Shimadzu, Japan) using Suprasil quartz cuvettes (Hellma Analytics, USA). The Raman spectrum at an excitation wavelength of 532 nm was recorded on an INVIA Microscopic Confocal Raman Spectrometer (Renishaw, England) equipped with a He–Ne laser. The infrared spectra were obtained on an Alpha-T spectrophotometer (Bruker, Germany) in the 4000–400 cm^–1^ range with the KBr pellet technique. A Quattro FEG-ESEM (FEI, USA) scanning transmission electron microscope was used to investigate the morphologies of ON–CDs. High-resolution transmission electron microscopy measurements were performed using a Talos F200S transmission electron microscope (FEI, USA) operated at 200 kV. X-ray photoelectron spectroscopy (XPS) of the ON–CDs was analyzed with a K-Alpha spectrometer (Thermo Fisher, USA). Time-resolved fluorescence measurements were collected using a Horiba Jobin Yvon SPEX Fluorolog 3-2iHR fluorescence spectrophotometer (Palaiseau, France). 3D-fluorescence mapping was recorded in matched Suprasil quartz cuvettes (Hellma Analytics, USA) and excitation–emission matrix (EEM) analysis was performed with a Quadro-II spectrofluorometer (Optical Building Blocks Co., USA). Dynamic light scattering (DLS) experiments were performed on a 90Plus particle size analyzer (Brookhaven Instrument, USA) using a 35 mW HeNe laser at a temperature of 25.0 ± 0.2 °C in water. A Seven Compact S220 pH-meter (Mettler Toledo, Switzerland) was used to perform all pH measurements. The magnetic stirring, centrifuge, and ultrasonic bath of the solutions were carried out on HSM 250D heating mantles with magnetic stirring (Thermomac, Germany), a Hettich Universal 320 centrifuge (Germany), and a WiseClean ultrasonic bath (Wisd, Germany), respectively. Drying processes for obtained ON–CDs were carried out in a WiseVen vacuum oven. A LC-20A liquid chromatograph (Shimadzu, Japan) coupled with an LCMS-8040 mass spectrometer (Shimadzu, Japan) was used for the proposed method validation. Liquid chromatography was equipped with an Ultra-AQ column (Restek, USA – 100 × 2.1 mm, 3 μm, C18).

### Reflux Synthesis of ON–CDs

ON–CDs were synthesized with a reflux method in the literature.^26^ CA anhydride (0.5 g, 2.60 × 10^–3^ mol), Glu (0.2 g, 1.10 × 10^–3^ mol), and EDA (75 μL, 1.10 × 10^–3^ mol) were dissolved in ultrapure water (10.0 mL) in a three-neck reaction flask. After that, the reaction flask was put inside a mantle heater and heated for 3 h at 180 °C while being stirred under reflux under an inert atmosphere (N_2_ gas). The brown solution was dialyzed in a cellulose membrane bag (MWCO 3500) for 36 h after cooling to room temperature (25 °C). Following the dialysis procedure, the solution was centrifuged at a high speed at 12,000 rpm, and the precipitate that was produced was dried in a vacuum oven at a temperature of 70 °C. Blue-emission ON–CDs were obtained as a brown solid. The ON–CDs (0.01 g mL^–1^) prepared in ultrapure water were used in experimental studies.

### Preparation of Solutions

The preparation of each solution used throughout the experiments is provided in the Supporting Information.

### Fluorescence Detection Procedure of PA

The sensitive detection of PA was performed with a spectrofluorometer. First, a stock solution of ON–CDs (0.01 g mL^–1^) was prepared in ultrapure water. Then, ON–CDs solution (300 μL), HEPES-Tris buffer solution (200 μL, 1.0 × 10^–2^ M, pH 7.0), and different amounts of PA solution (10 to 150 μL, 2.0 × 10^–7^ M) were added to a series of Eppendorf tubes. The final volumes of the solutions were made up to 2.0 mL with an ethyl alcohol (EtOH)–H_2_O (1:1, v/v) solvent mixture. The solution was allowed to stand for a minute to enable complete interaction between ON–CDs and PA. The fluorescence measurements were carried out between the emission range of 300 and 600 nm (λ_ex_ = 355 nm). All experimental trials were performed at room temperature.

### Selectivity and Interference Studies

Selectivity study for the determination of PA was performed in the presence of various types of explosives and their hydrolyzates (TNT, RDX, HMX, tetryl, PETN, NTO, and H_2_O_2_). Accordingly, ON–CDs solution (300 μL, 0.01 g mL^–1^) and HEPES-Tris buffer solution (200 μL, 1.0 × 10^–2^ M, pH 7.0) were added to a series of Eppendorf tubes. Afterward, the test solutions (100 μL, 2.0 × 10^–7^ mol L^–1^, PA, TNT, RDX, HMX, tetryl, PETN, NTO, and H_2_O_2_) were added separately to the Eppendorf tubes with ON–CDs solution and HEPES-Tris buffer solution. The final volumes of the mixtures were made up of 2.0 mL with an EtOH–H_2_O (1:1, v/v) solvent mixture. After allowing 1 min for ON–CDs to interact with explosives, the fluorescence emission spectra of all prepared solutions were recorded in the wavelength range of 300–600 nm (λ_ex_= 355 nm).

In addition, the accuracy of PA determination in the presence of possible interferences, such as selected metal cations (Fe^2+^, Cd^2+^, Mg^2+^, Mn^2+^, Cu^2+^, Zn^2+^, and Ca^2+^), common anions (Cl^–^, SO_4_^2–^, CO_3_^2–^, NO_3_^–^, and NO_2_^–^), explosives {TNT, tetryl, HMX, RDX, PETN, H_2_O_2_, NTO, and composite B (comp B contain TNT and RDX mixture)}, and camouflage materials (detergent, glucose, aspartame, paracetamol, and acetylsalicylic acid), was investigated. Potential interferents (metal cations and anions at 100-fold; 100 μL; 2.0 × 10^–5^ M, explosives at 20-fold; 100 μL; 2.5 × 10^–5^ M, and camouflage materials at 25-fold; 100 μL; 5.0 × 10^–6^ M) were added to vials containing stock ON–CDs (300 μL; 0.05 g mL^–1^) and HEPES-Tris buffer (200 μL; 1.0 × 10^–2^ M; pH 7.0), and PA (100 μL; 2.0 × 10^–7^ M) was added to each vial. Final volumes of the solutions were made up to 2.0 mL with an EtOH–H_2_O (1:1; v/v) solvent mixture. Fluorescence emission spectra of the prepared solutions were recorded in the wavelength range of 300 to 600 nm (λ_ex_= 355 nm).

### Calculating the QY

The QY of ON–CDs was measured by comparing the fluorescence intensities and absorption values of ON–CDs samples with anthracene (QY 0.27,^27^ dissolved in EtOH, excitation wavelength: 365 nm). In order to minimize the reabsorption effect, the absorbance of ON–CDs samples was kept below 0.1. The QY of ON–CDs was measured according to
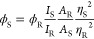
1where ϕ was the quantum yield, *I* was the measured integrated emission intensity, *A* was the absorbance measured on the ultraviolet–visible spectrophotometer, and η is the refractive index. The subscript “S” and “R” refers to ON–CDs and reference standard substance (the refractive index of water was 1.33 and of alcohol was 1.36), respectively. The QY of ON–CDs found in eq 1 was 49.7%. All of the obtained parameters are summarized in Table S1.

### PA Analysis in Soil Samples

As a certified reference standard, 2.0 g of clean sandy soil (uncontaminated soil no. 1, CLN SOIL-1) was weighed and transferred to a beaker. The sandy soil in the beaker was artificially contaminated with a solution of PA prepared in acetone solvent (2.5 mL; 4.50 × 10^–3^ M). The soil sample contaminated with PA was homogenized in an ultrasonic bath for 5 min and dried in a vacuum oven at 50 °C. For back-extraction of PA from sandy soil, the dried soil sample was washed with 2.0 mL of MeOH and the heterogeneous solution was centrifuged (10,000 rpm, 5 min). The supernatant was filtered (CHROMOFIL PET-20/25) into a 25.0 mL flask, and the final volume of the solution was made up to 25.0 mL with MeOH. This process was repeated three times for five clean sandy soil samples. The PA extract solution was diluted 1250 times, and PA was determined by the developed method.

The literature method involving liquid chromatography coupled to tandem mass spectrometry (LC–MS/MS) was applied to validate the proposed PA detection method.^28^ All experimental details of the reference LC–MS/MS method are provided in the Supporting Information.

### Preparation of Polymer Film-Based Support Material for PA Detection

The PF(ON–CDs) (PVA/ON–CDs) polymer films were prepared as support material for the practical application of ON–CDs. First, PVA granules (2.0 g) were dispersed in ultrapure water (20.0 mL) with continuous mixing. The prepared mixture was heated to 90 °C, and then the mixture was kept at this temperature for an additional 1 h for complete dissolution of the PVA. Afterward, 3.0 mL of ON–CDs solution (0.01 g mL^–1^) was added to the dissolved PVA solution and stirred for an additional 30 min. Finally, the resulting slightly yellow solution was transferred to a clean Petri dish (5.0 × 5.0 cm^2^) and dried in a vacuum oven at 45 °C for 12 h. After drying, the PF(ON–CDs) polymer films were peeled off of the Petri dish.

## Results and Discussion

### Synthesis and Photophysical Properties of ON–CDs

**Scheme 1:**
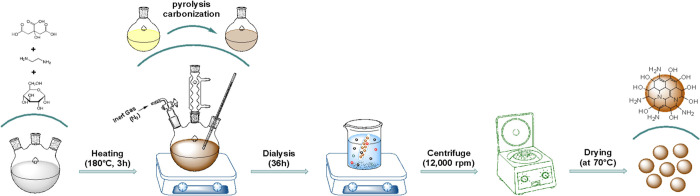
Representative Scheme Showing ON–CDs Synthesis

ON–CDs were manufactured by pyrolysis of molecular precursors under an inert atmosphere.^26^ Briefly, CA (as carbon source), D-Glu (as oxygen source), and EDA (as nitrogen source) were added to the three-necked reaction flask, and all molecular precursors were dissolved in 10.0 mL of ultrapure water. As shown in Scheme 1, the molecular precursors mixed in the reaction flask were refluxed under the optimized conditions. During this time, surface functionalization takes place by pyrolysis and subsequent carbonization of molecular precursors.^29^ At the end of these stages, ON–CDs were obtained in clusters with the carbonized polymer layer forming the core and the Lewis-basic groups functionalized on the surface (−OH, −NH_2_). However, since every polymeric carbon layer formed would not be of the same size, the synthesized ON–CDs were taken to a membrane bag made of cellulose (MWCO 3500) and dialysis was conducted against H_2_O for 36 h to obtain a monodisperse solution. At the end of this period, the solution remaining in the dialysis membrane was centrifuged at high speed (12,000 rpm), and the obtained precipitate was vacuum-dried in an oven at 70 °C.

**Figure 1:**
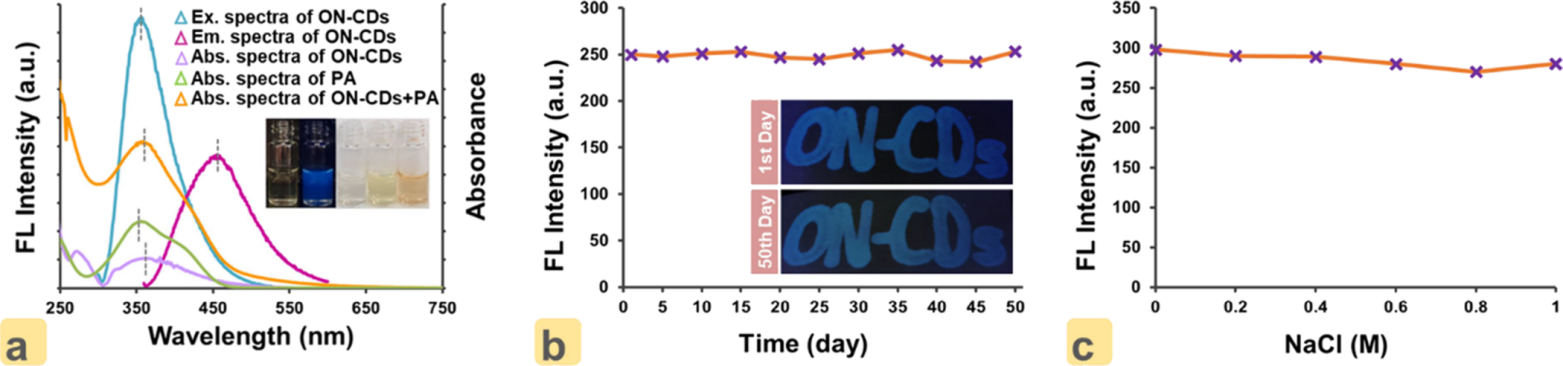
(a) Fluorescence excitation/emission spectra of ON–CDs, and the absorption spectra in the UV–vis region of ON–CDs, PA, and ON–CDs complex with PA, respectively (the inset shows ON–CDs solution, PA solution, and mixture solution of ON–CDs with PA under daylight and UV-light at λ: 365 nm). (b) Measurements for photostability of the ON–CDs solution (0.01 g mL^–1^) (the inset shows the handwritten (ON–CDs) on filter paper under 365 nm excitation with a UV lamp after 1 day (top photograph) and 50 days (bottom photograph) of storing time). (c) Intensity of fluorescence of ON–CDs at different NaCl concentrations in an aqueous medium.

Photophysical properties of ON–CDs were studied by absorption and fluorescence spectroscopies. Figure 1a displays the excitation/emission spectra of ON–CDs and the absorption spectra of ON–CDs, PA, and ON–CDs with PA, respectively. The ON–CDs solution, which is almost colorless in daylight, assumes a bright blue color under UV-light (λ_ex_= 365 nm) (Figure 1a-inset). ON–CDs exhibit the strongest fluorescence at 455 nm when excited at 355 nm. Inspecting the absorption spectrum of ON–CDs reveals two peaks centering at 275 and 355 nm. The peak noticed at 275 nm wavelength can be assigned to the π–π* transitions of the sp^2^ hybrid (i.e., C=C) graphite layer of ON–CDs.^30^ The peak at 355 nm may arise from the *n*–π* transition in the bonds between heteroatoms existing (i.e., C=O and C=N) on the carbon atom of ON–CDs.^31^ In addition, the absorption band peak (at 355 nm) and the excitation band peak (at 355 nm) of the ON–CDs completely overlap. In addition to ON–CDs, the absorption spectrum of the target analyte, PA, is also included. The 350 nm peak absorption band of PA, which displays its characteristic yellow color in daylight (Figure 1a-inset), mostly overlaps with the excitation band and partially overlaps with the emission band of ON–CDs. However, the color of the ON–CDs solution containing PA in daylight is orange, unlike the characteristic colors of PA, which is yellow, and that of ON–CDs, which is almost colorless (Figure 1a**-**inset). The absorption band (at 360 nm) of the ON–CDs after interaction with PA solution (i.e., of ON–CDs + PA) highly overlaps with the excitation/emission bands of ON–CDs (Figure 1a).

To see the photostability of ON–CDs in ultrapure water dispersion, the intensity of emission for the solution (0.01 g mL^–1^) at λ = 455 nm was daily recorded. After every measurement, the ON–CDs solution was kept at 5 °C. It is clearly visible from Figure 1b that the intensity of the fluorescence of the ON–CDs solution remained constant for 50 days. The excellent photostability of the ON–CDs solution for 50 days was confirmed with recoveries obtained between 98.1 and 102.4%. This confirms that the prepared ON–CDs solution can be used for up to 50 days. Additionally, a certain part of the same ON–CDs solution was taken to a clean blank pen, and the “ON–CDs” was handwritten on paper as observed in Figure 1b-inset. The photograph of the filter paper was taken under a UV lamp (λ_ex_ = 365 nm). For reproducibility, the same paper specimen was resubjected to the 365 nm UV lamp after 50 days elapsed and photographed. It was understood that ON–CDs, which maintained stable fluorescence in the solution medium in both photographs, showed the same performance on filter paper.

As described in Figure 1c, the intensity of fluorescence of ON–CDs at different NaCl concentrations (0.2–1.0 M) did not show significant variations, proving that NaCl did not notably influence the observed stability of ON–CDs. On the other hand, the critical coagulation concentrations of several carbon-based nanoparticles remain below 160 mM, typically at several tens of millimolar NaCl.^32^ The comparison of properties of synthesized oxygen- and nitrogen-doped carbon quantum dots (ON–CDs) with those of different carbon dots available in the literature is given in Table S2.

### Morphology and Surface Structure Characterization of ON–CDs

**Figure 2:**
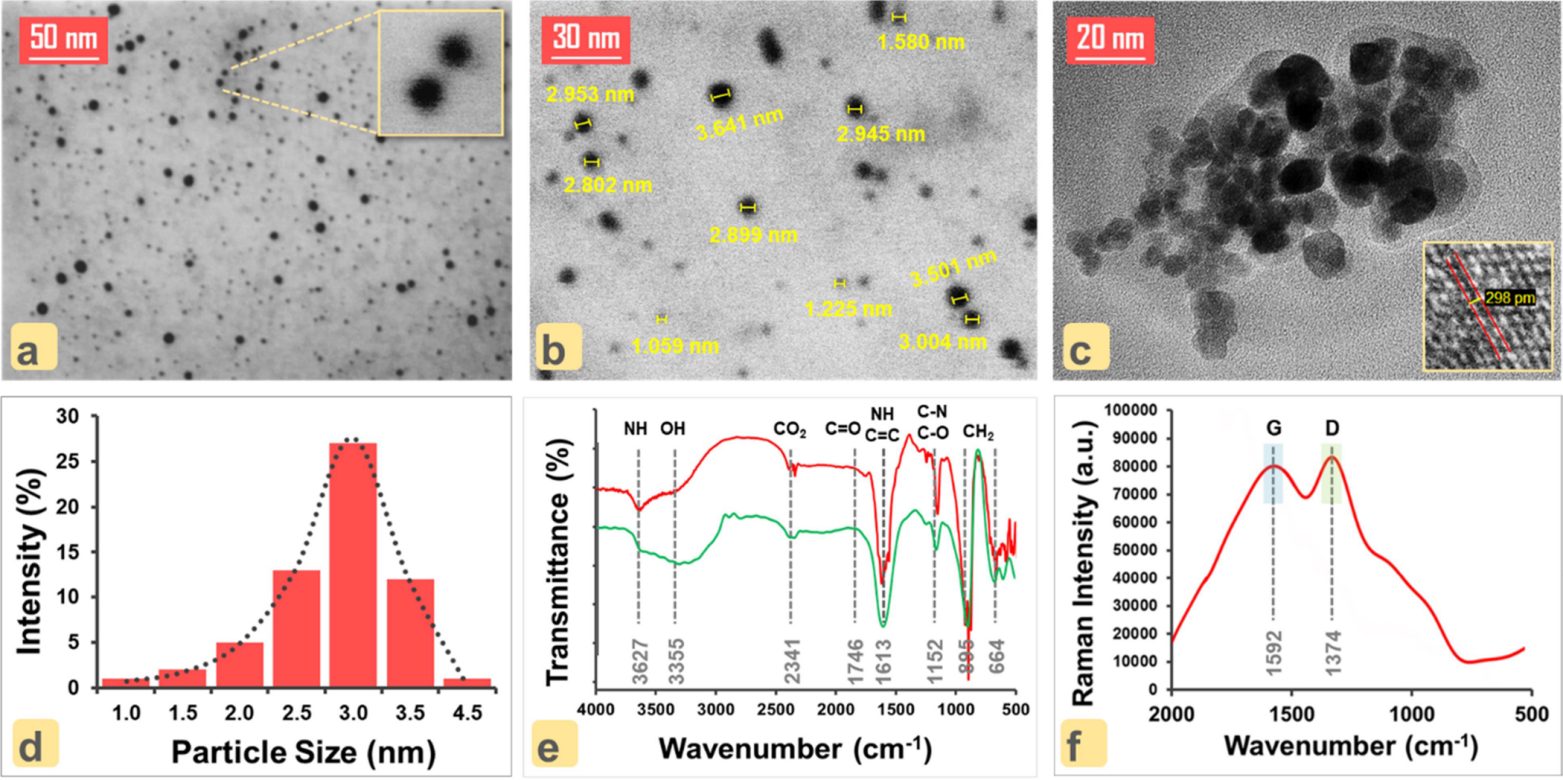
STEM images of ON–CDs at (a) 50 nm scale and (b) 30 nm scale. (c) HRTEM image of ON–CDs at 20 nm scale (inset: typical lattice constant parameter of ON–CDs). (d) Size distribution histogram of ON–CDs by DLS. (e) Infrared (KBr pellet) spectra of ON–CDs (red line) and ON–CDs + PA (green line). (f) Raman spectra of ON–CDs.

Surface morphology and size characterization of ON–CDs were performed using different instrumental techniques (STEM, DLS, IR, Raman, and XPS). First, the imaging of ON–CDs was carried out with scanning transmission electron microscopy (STEM) and high-resolution transmission electron microscopy (HRTEM). When the STEM images shown in Figure 2a (at 50 nm zoom) and b (at 30 nm zoom) and the HRTEM image shown in Figure 2c (at 20 nm zoom) are examined, it is seen that the oxygen- and nitrogen-doped carbon quantum dots (ON–CDs) have an almost spherical (ellipsoidal) structure. Additionally, 3D models (virtual thermal LUT) of TEM images were employed with the use of ImageJ software (Figure S1 for Figure 2c). This 3D model allows the particle distribution of ON–CDs to be better visualized on a magnified scale. However, it is understood that the ON–CDs have different sizes. DLS technique was used to generate the size distribution plot of ON–CDs. In the graph given in Figure 2d, it can be visualized that the sizes of ON–CDs range from 1.0 to 4.5 nm and the average particle size is 3.0 (±0.5) nm. This confirms that ON–CDs show monodisperse distribution in the solution medium.

Infrared spectroscopy was used to confirm the surface functionalities of the ON–CDs after imaging and dimensional analysis. In the infrared (IR) spectrum of ON–CDs, a characteristic broad peak appears around 3300 cm^–1^, indicative of the high percentage of hydroxyl (−OH) groups on the surface. When the IR spectrum given in Figure 2e (red line) is examined, the expected −OH band is seen at 3355 cm^–1^. In addition, the stretching vibration peak of the amine (N–H) group on the ON–CDs is observed at 3627 cm^–1^. The double peak seen around 2340 cm^–1^ (2345 and 2338 cm^–1^) can be assigned to CO_2_ gas absorbable from air.^33^ The characteristic peak of the functionalized C=O (stretching) group of the polymer carbon layer is observed at 1746 cm^–1^, and the peaks observed at 1613 and 1599 cm^–1^ represent N–H (bending) and C=C (stretching) vibrations, respectively. The characteristic band of amide carbonyl (arising from the cross-linking of EDA with CA^34^) emerges at 1638 cm^–1^. The peaks seen around 1150 cm^–1^ (1153 and 1145 cm^–1^) are C–O and C–N bending vibrations indicating the heteroatom functionalization of graphite.^35^ C–H (−CH_2_) bending vibrations can be assigned to the binary peaks observed at 895 and 660 cm^–1^. The degree of graphitization of the ON–CDs was investigated by Raman spectroscopy. The peaks observed at 1374 and 1592 cm^–1^ in Figure 2f are the characteristic peaks of the D- and G-bands, respectively. Of the scattering bands seen here, D-explains the graphite void defects of sp^3^-hybrid amorphous carbon and G- explains the graphene layer of sp^2^-hybrid carbon atoms.^36^ In addition, the quotient of the intensities of D- to G-bands (ID/IG) of defective carbon atoms can give an idea of the extent of graphitization. The ratio of ID/IG of the carbon dots used in this work is 1.01, which confirms the high extent of defects on the ON–CDs surface.^37^

**Figure 3:**
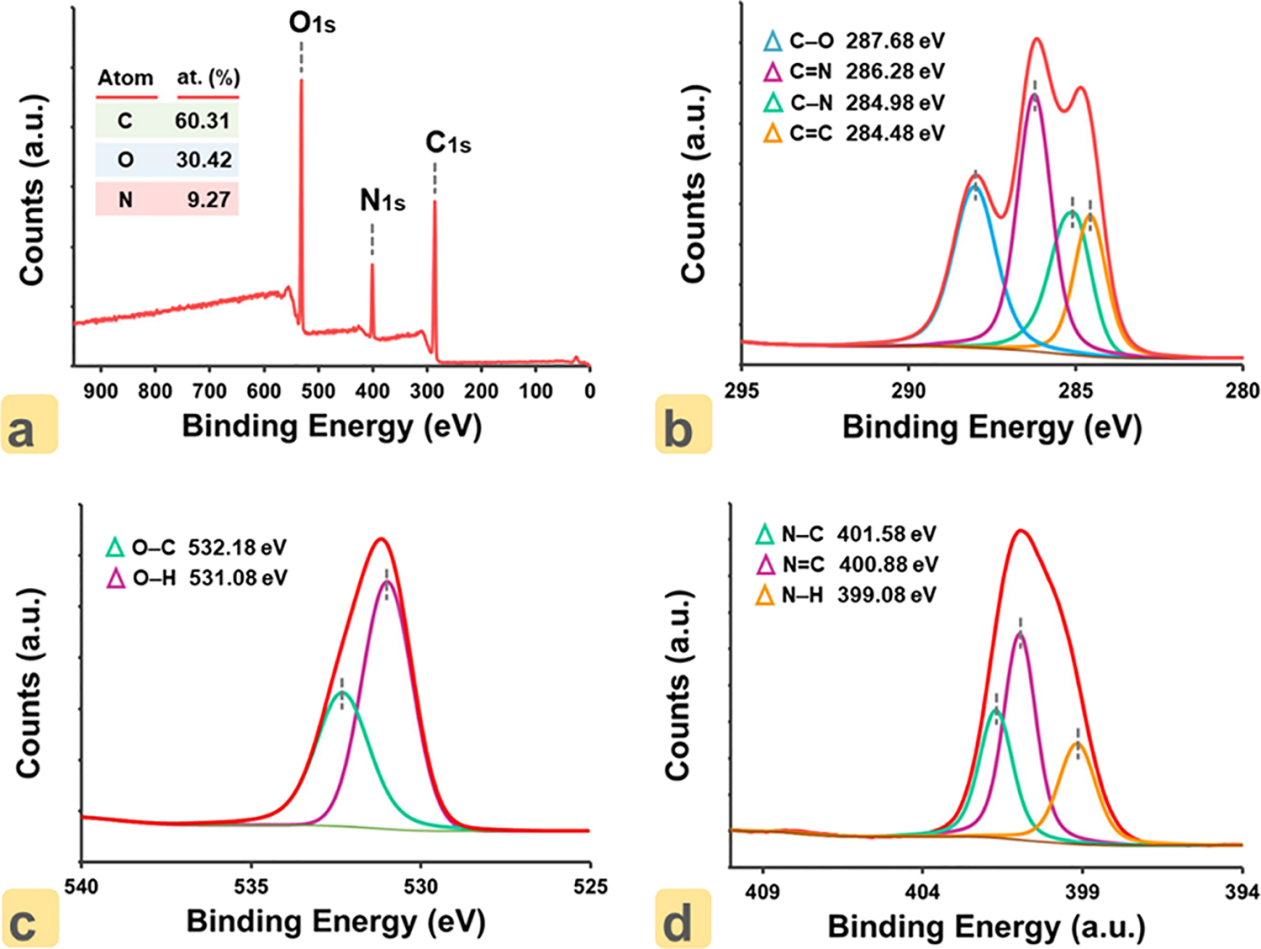
(a) XPS survey spectra of ON–CDs. High-resolution spectra of (b) C 1s, (c) O 1s, and (d) N 1s.

XPS analysis was performed to better elucidate the structure of ON–CDs. When the overall scanning spectrum of XPS given in Figure 3a is examined, characteristic peaks of C 1s, N 1s, and O 1s are seen at 286, 400, and 500 eV, respectively. These data yielded the contents of C, O, and N elements in the sample as 60.31, 30.42, and 9.27%, respectively. The relatively high carbon and oxygen contents of ON–CDs as a carbon-based material bearing surface-functionalized hydroxyl groups is predictable. A high-resolution scan of the C 1s spectrum of ON–CDs is shown in Figure 3b. Although the spectrum contains four different peaks, it is known that the first of these peaks is the one supporting the graphitic structure (sp^2^, C=C) at 284.48 eV. The other peaks at 284.98, 286.28, and 287.68 eV can be attributed to the sp^3^ carbon (C–N) single-bonded to nitrogen, the sp^2^ carbon (C=N) double-bonded to nitrogen, and the oxygen–carbon bond (C–O), respectively.^38^ In Figure 3c, it is seen that there are two peaks in the O 1s spectrum of ON–CDs where the bonds of oxygen with hydrogen atom (O–H) and oxygen with carbon atom (O–C) can be assigned to 531.08 and 532.18 eV binding energies, respectively.^39^ Finally, when the N 1s spectrum given in Figure 3d is examined, the three peaks seen at 399.08, 400.58, and 401.58 eV support the presence of N–H, N=C, and N–C bonds, respectively.

### Optimization of Experimental Conditions

The optimal parameters for ON–CDs pertaining to reaction temperature (Figure S2a,b), reaction time (Figure S3a,b), wavelength (Figure S4a,b), solvent system (Figure S5a,b), and medium-pH (Figure S6a–c) are as described in the Supporting Information.

### PA Detection Using ON–CDs

**Figure 4:**
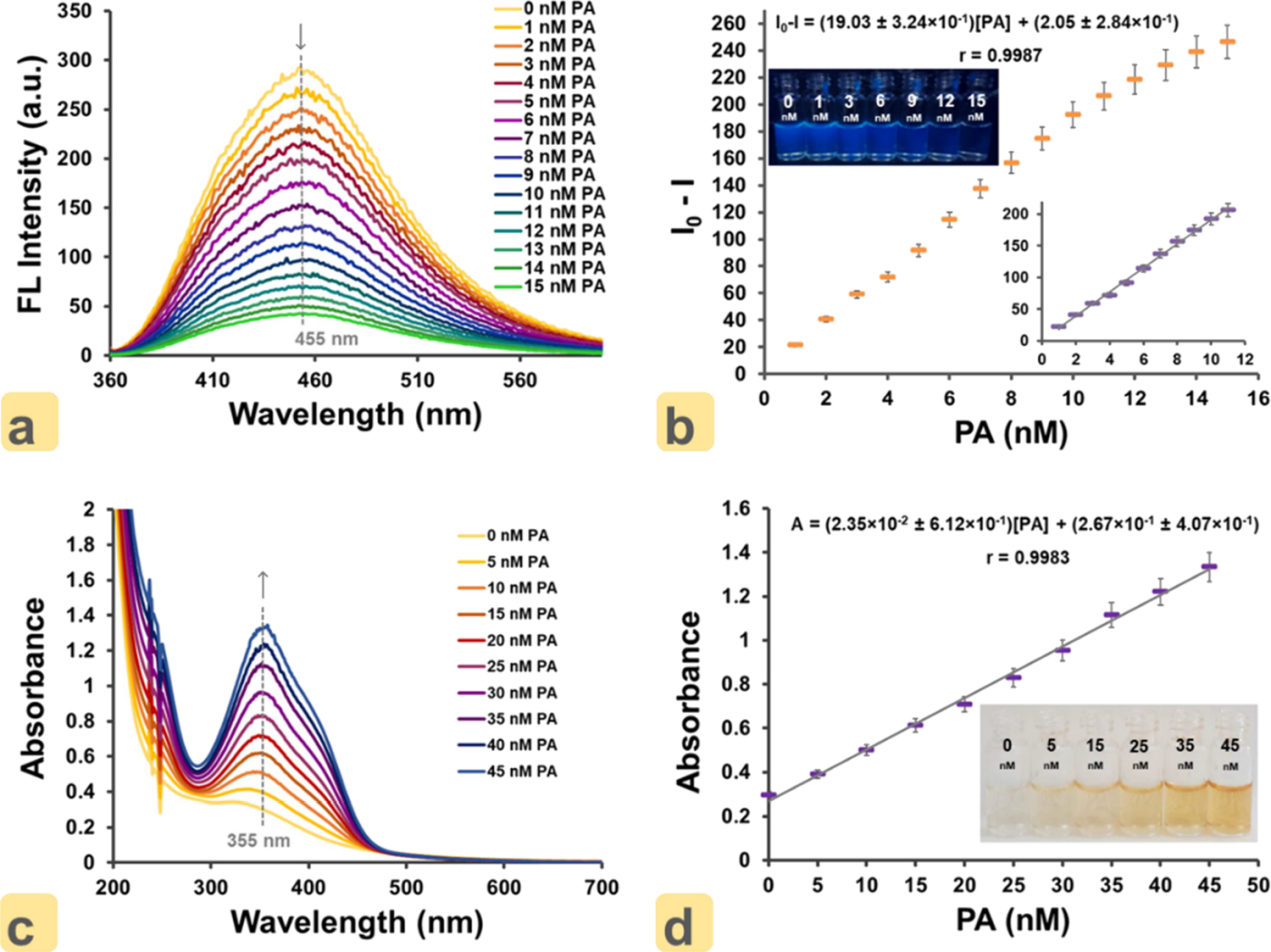
(a) Fluorescence emission spectra of ON–CDs at different PA concentrations (1.0 × 10^–9^–15 × 10^–9^ M, λ_ex_ = 355 nm). (b) Relationship between intensity difference (*I*_0_–*I*) and increasing PA concentrations, where *I*_0_ and *I* are the fluorescence intensities of ON–CDs in the absence and presence of PA, respectively (inset: photographs of ON–CDs after adding different amounts of PA under a UV lamp at 365 nm). (c) Absorption spectra of ON–CDs at different PA concentrations (5.0 × 10^–9^–45 × 10^–9^ M, λ_ex_ = 355 nm). (d) Relationship between *A* and increasing concentrations of PA, where *A* is the absorbance of ON–CDs in the absence (blank) and presence of PA (inset: photographs of ON–CDs taken after adding different amounts of PA under daylight).

In order to investigate the variation between the intensity of fluorescence of ON–CDs and the concentration of PA, the recording of the fluorescence spectrum of ON–CDs by adding PA under the predetermined experimental conditions was made. PA solutions at increasing volumes (10–150 μL from 2.0 × 10^–7^ M initial concentration) were added to a series of vials containing ON–CDs (300 μL, 0.01 g mL^–1^) and HEPES-Tris buffer solution (200 μL, 1.0 × 10^–2^ M, pH 7.0). The final volumes of the vials were diluted to 2.0 mL with an EtOH–H_2_O (1:1, v/v) mixture. All solutions were recorded for their fluorescence in the λ range of 300–600 nm (λ_ex_ = 355 nm). As shown in Figure 4a, it is seen that the emission band of the ON–CDs was quenched with increasing PA concentration. Fluorescence intensities recorded at 455 nm (i.e., emission maximum) wavelength were plotted against PA concentration, and a calibration curve was prepared (Figure 4b), showing that the change in the fluorescence intensity of ON–CDs had excellent linearity between 1.0 × 10^–9^ and 11.0 × 10^–9^ M concentrations of PA (Figure 4b**-**inset). In addition, in Figure 4b-inset, there is a photograph of ON–CD solutions taken under a UV lamp (λ = 365 nm) against increasing concentrations of PA. As with the emission bands recorded with increasing PA concentration, the fluorescence of ON–CDs is quenched (left to right). The linear variation of ON–CDs intensity decrement (Δ*I*) with PA nanomolar concentration (*C*_PA_) is represented with the equation:
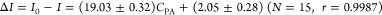
where *I*_0_ and *I* denote the intensities of fluorescence at 455 nm in the absence and presence of PA, respectively. The detection limit (LOD) for PA was found to be 12.5 × 10^–12^ M (12.5 pM), and the coefficients of variation (CV) were 0.97% (intra-assay) and 1.43% (inter-assay). The Δ*I* values of blank-level samples were used to calculate the standard deviation, which was subsequently multiplied by three and divided by the calibration line slope to compute the LOD.

With the addition of PA to the ON–CDs solution, the solution changes from colorless to orange color. The observed orange color confirms that a complex is formed between ON–CDs and PA, resulting in coloration, except for the fluorometric interaction. This leads us to investigate how the absorption band of ON–CDs will change with PA concentration. In this direction, increasing amounts of PA (50–450 μL from 2.0 × 10^–7^ M initial concentration) were added to the vials containing ON–CDs (300 μL, 0.01 g mL^–1^) and HEPES-Tris buffer solution (200 μL, 1.0 × 10^–2^ M, pH 7.0), and the final volume of the vials was completed to 2.0 mL with an EtOH–H_2_O (1:1, v/v) solvent mixture. UV–vis spectral recording in the wavelength range 200–700 nm was made for the prepared solutions. When the absorption bands given in Figure 4c are inspected, it is clearly seen that the absorbance of ON–CDs at 355 nm wavelength (i.e., absorbance maximum) increases smoothly with increasing PA concentration (5.0 × 10^–9^–45.0 × 10^–9^ M). By plotting the absorbance values against PA concentration at a 355 nm wavelength, a perfect linear relationship was obtained between the absorbance of ON–CDs and PA (Figure 4d). There are also photographs of the solutions of ON–CDs prepared with increasing amounts of PA, given in Figure 4d-inset. These photographs show that the resulting orange color (from left to right) increases with PA concentration. The equation for the linear variation of ON–CDs absorbance (*A*) with PA nanomolar concentration (*C*_PA_) is given as
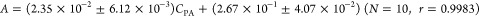
where *A* represents the 355 nm absorbance in the absence and presence of PA, respectively. As seen from the calibration equation, the ON–CDs without PA had an initial blank absorbance. The PA LOD was calculated to be 9.0 × 10^–10^ M (0.9 nM), and the CV were 1.29% (intra-assay) and 1.64% (inter-assay). The validation parameters regarding the performance of ON–CDs for PA detection are briefed in Table 1.

**Table 1:** Performance Parameters of the ON–CDs for Detection of PA

analyte	method	linear range^a^	LOD^b^	CV^c^ (%)	
				intra-assay	inter-assay
PA	fluorometric	1.0 × 10^–9^ – 11.0 × 10^–9^	12.5 × 10^–12^	0.97	1.43
	colorimetric	5.0 × 10^–9^ – 45.0 × 10^–9^	9.0 × 10^–10^	1.29	1.64
	smartphone	5.0 × 10^–6^ – 25.0 × 10^–6^	1.0 × 10^–6^	1.88	2.74

*a* In M units at final concentration.

*b* Limit of detection, in M units (LOD = 3 σ_bl/_*m*, σ_bl_ representing the standard deviation of a blank and *m* the slope of the calibration line).

*c* Coefficients of variation, as percentage (*N* = 5).

At this point, ON–CDs have three important advantages: (i) As given in Figure 1a-inset, the color of the PA solution is yellow, and this color remains stable in shades of yellow tones with increasing PA concentration. However, in the ON–CDs solution to which PA is added, orange color tones are observed, different from yellow. This is due to the interactions between ON–CDs and PA (i.e., hydrogen-bonding and charge-transfer interaction). The ability of ON–CDs to determine PA with more than one interaction (i.e., dual-mode detection) is an important advantage of manufactured ON–CDs. (ii) Analytical response is taken at the nanomolar (nM) level for the colorimetric analysis. In addition, these analytical responses can be recognized with the naked eye, which may be an advantage of ON–CDs in field colorimetric detections. (iii) Carbon dots-based probes available in the literature usually detect the analyte monofunctionally (i.e., fluorometrically),^40,41^ and the resulting fluorescence quenching may arise from different factors other than the analyte. However, the complex formation of ON–CDs with the target analyte by showing different interactions allows ON–CDs to determine the target analyte as dual-functional (i.e., both fluorometric and colorimetric), one confirming the other, thereby preventing false positive responses. Although the fluorometric mode has a much lower LOD, ON–CDs can also detect PA colorimetrically with sufficiently high sensitivity.

Additionally, this method was compared with other published literature methods regarding the determination of PA in terms of linear ranges, and LODs are listed in Table S3. Examination of Table S3 reveals that this fluorometric probe can yield a very low LOD and a reasonable range of linearity compared to other organic dye- and carbon dot-based fluorometric sensors.

### Fluorescence Quenching Mechanism for the Detection of PA

Fluorescence quenching mechanisms such as fluorescence resonance energy transfer (FRET; dynamic quenching mechanism) and inner-filter effect (IFE; static quenching mechanism) to detect different analytes have been well documented in the literature.^42^ To explore the current quenching mechanism of the proposed carbon dots-based fluorescence probe (ON–CDs), first, the optical properties (i.e., absorption spectra and fluorescence excitation/emission spectra of ON–CDs and ON–CDs + PA complex) were investigated. It has been well reported in the literature that the FRET process essentially occurs when the fluorescence emission spectra of the fluorophore overlap with the absorption spectra of the analyte or quencher.^43^ In the graph given in Figure 1a, it can be seen that the emission spectrum of ON–CDs does not overlap with the absorption spectrum of PA, suggesting the noninvolvement of FRET. If the analyte absorption spectrum (showing fluorescence quenching) overlaps with the fluorescent excitation/emission spectra of the fluorescent sensing substance (fluorophore), the analyte absorbs the excitation/emission radiation of the fluorophore with IFE, resulting in effective quenching of the sensor fluorescence. It is apparent that the excitation spectrum of ON–CDs given in Figure 1a significantly overlaps with the absorption spectrum of PA; this suggests that the IFE is the primary cause of the fluorescent quenching of the (ON–CDs+PA) complex. In addition, it is likely that there are other static quenching mechanisms in the present method besides IFE. Static quenching is usually induced due to the strong interaction of fluorophore and quencher and to the formation of a nonfluorescent ground-state complex,^44^ which was examined by UV–vis absorption spectra of ON–CDs, PA, and (ON–CDs + PA) system (Figure 1a). Here, it is seen that the absorbances of PA alone display values within the whole spectral range that are lower than the corresponding absorbances of the complex formed with ON–CDs. However, the orange color of ON–CDs + PA solution shown in Figure 4d-inset suggests ground-state complex formation between the fluorophore (ON–CDs) and quencher (PA) and supports static quenching.

**Figure 5:**
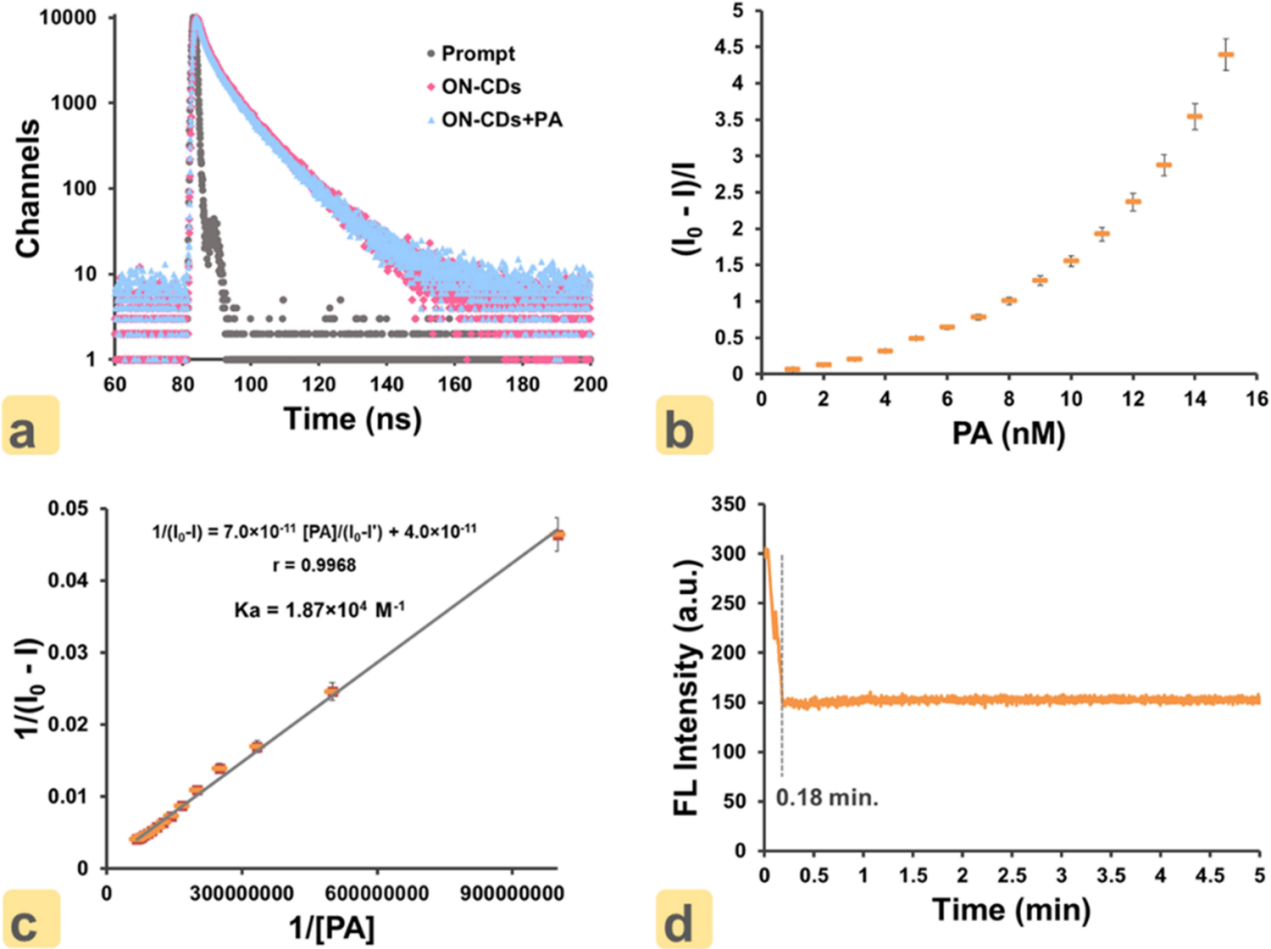
(a) Fluorescence lifetime decay curves of ON–CDs and ON–CDs + PA. (b) Stern–Volmer plot for fluorescence quenching of ON–CDs with varying concentrations of PA. (c) Benesi–Hildebrand plot of ON–CDs in the presence of PA. (d) Time-dependent fluorescence intensities of ON–CDs (0.01 g mL^–1^) recorded in the presence of PA (7 × 10^–9^ M).

The fluorescence lifetime decay curves of ON–CDs and the ON–CDs + PA complex given in Figure 5a were used to confirm the mechanism of static quenching. The mean fluorescence lifetime degradation of ON–CDs is measured as 6.85 ± 0.03 ns, while that of ON–CDs + PA complex is 6.91 ± 0.05 ns. The nearly constant mean fluorescence lifetime degradation of ON–CDs in the absence or presence of PA hints at ground-state complex formation between ON–CDs and PA, in favor of a static quenching mechanism. In addition, the absence of any linear intervals in the Stern–Volmer curve given in Figure 5b supports the static quenching mechanism. Another parameter supporting the static quenching mechanism is the Benesi–Hildebrand plot in Figure 5c. The association constant (*K*_a_) obtained by this linear interaction observed between ON–CDs and PA was found to be 1.87 × 10^4^ M^–1^. This value confirms the formation of a strong complex between the host (ON–CDs) and the guest (PA) molecule. In addition, when the time-based fluorescence intensity measurement graph in Figure 5d is examined, it is seen that 0.18 min passes for the quenching of fluorescence by adding PA to the ON–CDs solution, and the intensity of fluorescence remains constant for 5.0 min after quenching.

It is thought that there are two different interactions leading to the ground-state complex, i.e., hydrogen-bonding, and charge-transfer complexation (CTC). It is highly probable that there is a hydrogen-bonding interaction between the phenol group (Ar–OH) of PA and the hydroxyl (−OH) and amino (−NH_2_) groups of ON–CDs, and there is a CTC between the nitro (−NO_2_) groups of PA and the amino (−NH_2_) groups of ON–CDs. It is known that hydrogen-bonding interaction weakens in aqueous solution and is stronger in an organic solvent.^45^ In the proposed method, it is worked with an EtOH–H_2_O (1:1, v/v) solvent mixture. If our hypothesis is correct and if we work entirely with H_2_O in the method, then the detection sensitivity of ON–CDs to PA will decrease. Looking at the graphs given in Figures S7a,b, if the working solution is brought from 50 to 100% aqueous content, the fluorescence intensity of ON–CDs to detect PA decreases by half, confirming the presence of a ground-state complex via hydrogen-bonding interaction between ON–CDs and PA. In this case, CTC between ON–CDs and PA can be assumed to be of secondary importance in ground-state complex formation, as the electron-withdrawing effects of PA nitro groups are not as strong as those in TNT.

In order to confirm the ground-state complex formation between ON–CDs and PA, the infrared spectrum (Figure 2c-green line) of the ON–CDs + PA complex was taken as well as of ON–CDs. When the spectrum given in Figure 2e (green line) is examined, the −OH peak located at around 3355 cm^–1^ of the ON–CDs becomes broader in the presence of PA. This confirms that there is a hydrogen-bonding interaction in the resulting complex. It is observed that the intensity of the N–H stretching peak seen at 3627 cm^–1^ has decreased. Similarly, the N–H bending peak at 1613 cm^–1^ disappears in the presence of PA and the sharp peak becomes broader. In addition, the amide carbonyl peak at 1638 cm^–1^ almost disappears. All of these observed changes support ground-state complex formation between the amine (−NH_2_) groups of the ON–CDs and the hydroxyl (−OH---NH_2_–; hydrogen-bonding interaction) and nitro (−O_2_N^δ−^---^δ+^NH_2_; charge-transfer interaction) groups of PA. Additionally, in spectrophotometric tests, the appearance of a new colored product from the analyte and probe confirms CT interactions.

**Figure 6:**
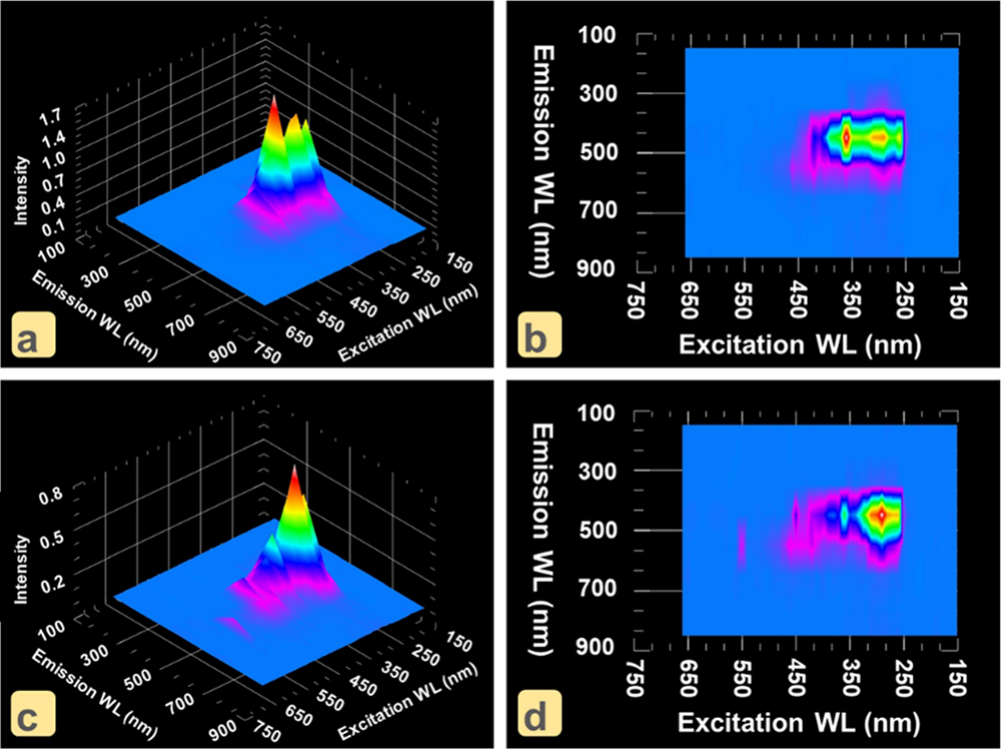
(a) 3D-fluorescence spectra and (b) EEM analysis of ON–CDs. (c) 3D-fluorescence spectra and (d) EEM analysis of ON–CDs + PA.

Regarding three-dimensional (3D) fluorescence spectroscopy, components such as inter/intramolecular interactions, conformation, structure, presence of target analyte, and heterogeneity of the resulting complex can be observed by excitation–emission matrix (EEM) analysis.^46^ Therefore, the fluorescence detection and interaction mechanisms of ON–CDs for PA were evaluated by EEM analysis and 3D fluorescence spectroscopy under optimized conditions. As given in Figure 6a,b, ON–CDs have two different excitation states in the ranges 200–300 and 300–400 nm. The excitation in the 250–350 nm range (excitation maximum: λ_ex_ at 280 nm) is due to the graphite layer of ON–CDs, and the excitation in the 350–400 nm range (λ_ex_ = 355 nm) is due to the surface functional groups of ON–CDs (includes nitrogen and oxygen atoms, which are fluorescence traps). The fluorescence intensities of emissions with λ_ex_ = 280 and λ_ex_ = 355 nm are 0.5 and 1.2, respectively, indicating that ON–CDs display strong fluorescence. In Figures 6c,d, there is a 3D-fluorescence map of the EEM analysis of the ON–CDs + PA complex formed by adding PA to the ON–CDs solution. Examination of EEM showed that the fluorescence emission intensity of the λ_ex_: 355 nm peak decreased from 1.2 to 0.2, while the intensity of the λ_ex_: 280 nm peak did not change and remained at 0.5. The EEM analysis graphs in Figure 6 show that PA interacts with the surface functionalities of ON–CDs and not with the graphite layer, supporting the static fluorescent quenching mechanism with the proposed IFE and ground-state complex formation (resulting from hydrogen-bonding and charge-transfer interactions).

**Scheme 2:**
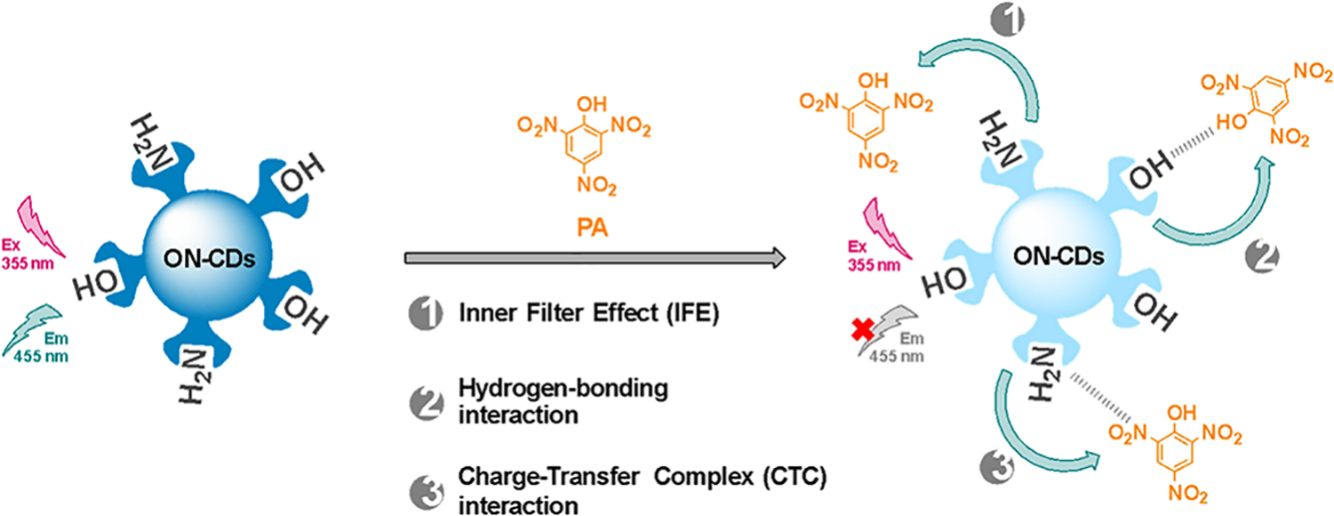
Illustration of PA Determination Depending on Fluorescence Quenching of ON–CDs

All of these analytical results lead to Scheme 2 summarizing the proposed fluorescence quenching mechanisms in the ON–CDs-based fluorometric method for the determination of PA.

### Selectivity Assay of ON–CDs for PA and Effect of Possible Interferences

The selectivity of ON–CDs toward the target analyte PA was investigated in the presence of potentially interferent explosive compounds and their conversion products in the nitroaromatic (TNT and tetryl in the same group as PA), nitramine (RDX and HMX), nitrate ester (PETN), peroxide (H_2_O_2_), and insensitive (NTO) groups. Detailed structural and molecular weight information on the tested explosives is summarized in the Supporting Information. As seen in Figure S8a (fluorescence spectra) and Figure S8b (intensity histogram), the fluorescence of ON–CDs is perfectly quenched in the presence of PA. This can be justified by the selectivity of the analytical assay proposed for PA, through the current IFE mechanism. In addition, other explosives have a small effect. There is a high probability of CTC between the nitro groups in the structure of these explosives and the amino groups of ON–CDs. Although the proposed method mainly relies on the IFE and hydrogen-bonding interaction, a weak CTC interaction was also postulated to take part. Had this charge-transfer interaction been of primary importance, TNT bearing stronger electron-withdrawing −NO_2_ groups should interfere with the proposed assay, which is not the case. According to the result obtained from the selectivity study, the CTC interaction was very weak, and ON–CDs showed excellent selectivity to PA.

In addition, explosive traces may exist either in contaminated land bearing a postblast debris or in explosive-polluted soil subjected to land remediation. Explosives other than PA do not show the capability of fluorescence quenching of ON–CDs due to their inability to form ground-state complexes with ON–CDs. Looking at the values given in Table 2, it is visible that other explosives or hydrolyzates (TNT, tetryl, RDX, HMX, PETN, H_2_O_2_, and NTO) that can be found together with PA do not have a significant effect up to 20-fold on the probe selectivity for PA. Recovery values are between 93.3 and 104.8%, which definitely shows the ability of the developed method to distinguish PA from other explosives.

**Table 2:** Recovery (%) Values of PA (at 10.0 × 10^–9^ M Final Concentration) from Explosive Mixtures

explosive mixture	mass ratio (PA/other explosive)	recovery (%)
PA + TNT	1:20	104.8
PA + tetryl	1:20	105.2
PA + HMX	1:20	93.3
PA + RDX	1:20	101.6
PA + RDX (comp B)	1:20	98.1
PA + PETN	1:20	99.6
PA + H_2_O_2_	1:20	97.4
PA + NTO	1:20	102.9

Finally, PA was added to the vials containing metal cations and common anions at 100-fold (of PA concentration) and camouflage materials at 25-fold (of PA concentration) to determine the interference effects on the selective detection of PA. Fluorescence spectra of each solution were recorded in the wavelength interval of 300–600 nm (λ_ex_ = 355 nm). Examining Figure S9a (fluorescence spectra) and S9b (intensity histograms) shows that ON–CDs without PA (pink bars for Figure S9b) and with PA (blue bars for Figure S9b) performed as expected, and potential interferents did not adversely affect the determination of PA. When these interferents were present, fluorescence recovery of ON–CDs without PA ranged between 94.6 and 105.4% and fluorescence recovery of ON–CDs with PA ranged between 95.2 and 102.5% (Table S4).

### Application of ON–CDs to PA Detection in Soil Sample

For the determination of PA in soil extract, artificial contamination of 2.0 g of clean sandy soil was made with 2.5 mL of acetone solution of PA, and solvent removal from the sandy soil was accomplished by keeping it in a vacuum oven at 50 °C. Then, PA was extracted with MeOH and a working solution was prepared with appropriate dilutions. The PA solutions extracted from the soil were subjected to the procedure given in the *Fluorescence Detection Procedure of PA*, and the concentration value of each PA solution was calculated. This process was applied to five different samples of soil. The resulting solutions of PA were analyzed using the proposed fluorometric method with good recoveries between 96.9 and 104.5%. The mean recovery from five different samples of soil was 101.2%. The average value of PA concentration in five different soil samples analyzed with the proposed fluorometric method is 3.57 × 10^–6^ M.

### Validation of the Spectrofluorometric Assay Against the Reference LC–MS/MS Technique

LC–MS/MS as a reference technique was used to analyze PA extracted from five sandy soil samples containing identical amounts of PA.^28^ First, the prepared working solutions (0.44 × 10^–6^, 0.87 × 10^–6^, 1.75 × 10^–6^, 3.5 × 10^–6^, and 4.4 × 10^–6^ M) were analyzed with LC–MS/MS by drawing a calibration line of which the equation is given below:
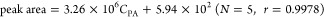


The sandy soil extracts were analyzed by LC–MS/MS, and the corresponding concentrations were found by the above equation. Statistical comparison of PA values found using both (proposed and reference) methods are shown in Table 3.

**Table 3:** Statistical Comparison of the Proposed Spectrophotometric Method with the LC–MS/MS Technique for PA-Contaminated Soil Sample Analysis

analyte	method	mean conc. (M)	SD (σ)	*S*^a^^,^^b^	*t*^a^^,^^b^	*t*_table_^b^	*F*^b^	*F*_table_^b^
PA	proposed fluorometric method	3.57 × 10^–6^	1.090					
	LC–MS/MS	3.48 × 10^–6^	0.904	1.992	0.965	2.306	1.166	6.39

*a* *S*^2^ = ((*n*_1_ – 1)*s*_1_^2^ + (*n*_2_ – 1)*s*_2_^2^)/(*n*_1_ + *n*_2_ – 2) and *t* =/(*S*(1/*n*_1_ + 1/*n*_2_)1/2), where *S* is the pooled standard deviation, *s*_1_ and *s*_2_ are the standard deviations of the two populations with sample sizes of *n*_1_ and *n*_2_, and sample means of ®_1_ and ®_2_ respectively (*t* has (*n*_1_ + *n*_2_ – 2) degrees of freedom); here, *n*_1_ = *n*_2_ = 5.

*b* Statistical comparison made on paired data produced using the suggested and reference methods; results being given only on the row of the reference method.

### PA Detection Using Polymer Film-Based Support Material

**Scheme 3:**
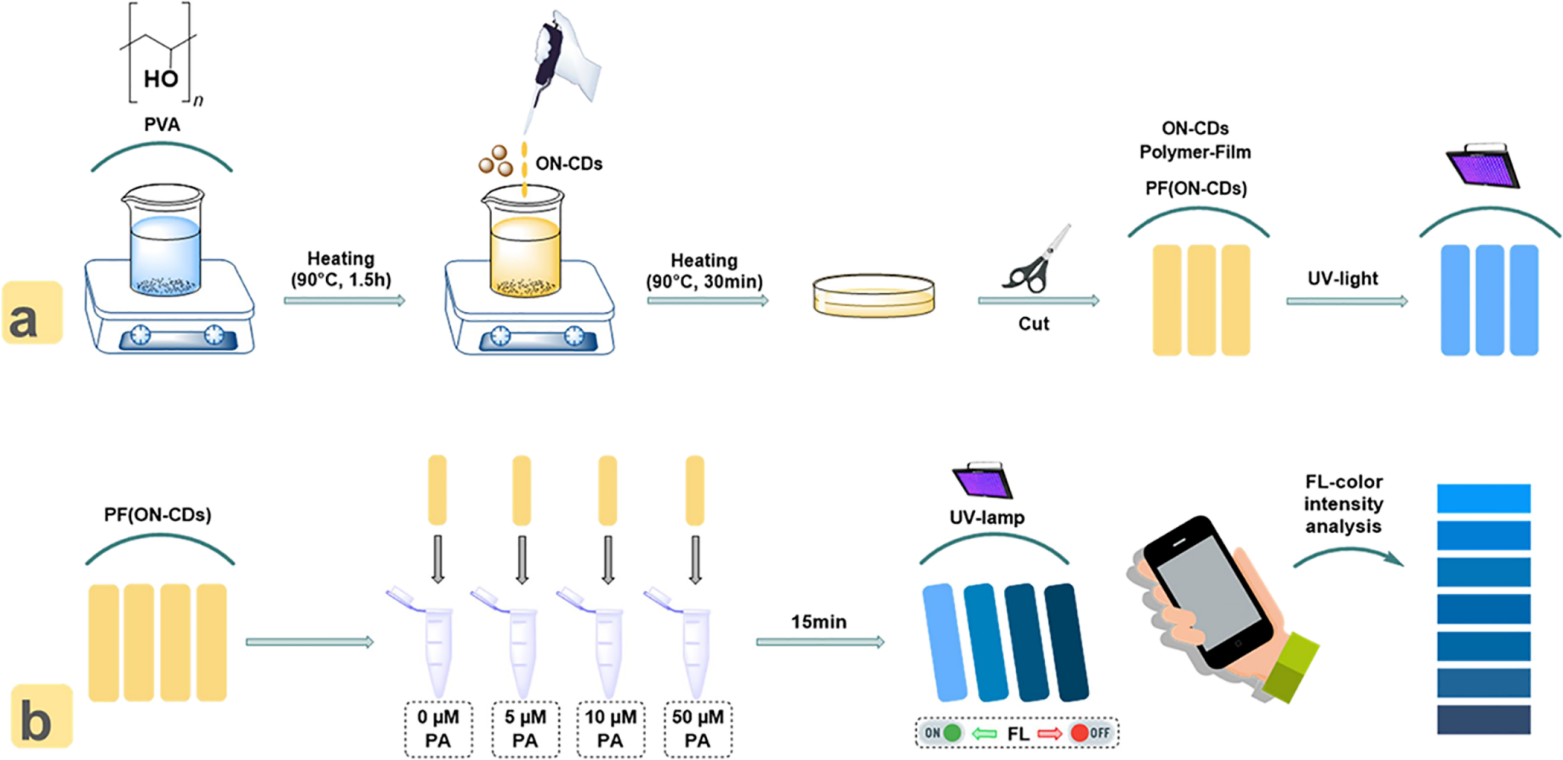
Schematic Representation of (a) Preparation of the PVA-Based ON–CDs Polymer Film {PF(ON–CDs)} and (b) Application of the PVA-Based ON–CDs Polymer Film {PF(ON–CDs)} to Different Concentrations (5.0 × 10^–6^–50 × 10^–6^ M) of PA

A PVA-based polymer film support material was developed for the practical application of ON–CDs to diverse media with high sensitivity and selectivity for PA. Typically, the PVA granules were taken into a beaker and completely dissolved in hot ultrapure water with stirring, as shown in Scheme 3a. At this stage, ON–CDs solution was added to hot PVA solution and stirring was continued for a certain time in order to homogeneously disperse the ON–CDs in the solution. Then, the prepared PVA-ON–CDs solution was taken into a Petri dish and dried in a vacuum oven. The PF(ON–CDs) removed from the Petri dish is an almost colorless, transparent material. As seen in Figure S10a–f, the flexible polymer {i.e., PF(ON–CDs)} can be folded, and the color of the film becomes yellow as the folding continues. The PF(ON–CDs) has a thickness of 1.0 mm and elastically returns to its original shape after folding.

Figure S11 shows images under a UV lamp at 365 nm of the polymer film (a) without and (b) with ON–CDs. The polymer film without ON–CDs does not show any fluorescence under a UV lamp, while the ON–CDs-containing polymer film, PF(ON–CDs), shows excellent fluorescence under UV. It is seen in the photograph that the fluorescence is all over the polymer film, confirming that the ON–CDs are homogeneously distributed on the polymer film. XPS survey spectra of the PVA-based polymer film and the ON–CDs-containing PVA-based polymer film {PF(ON–CDs)} were taken for a more detailed examination of the elemental content of the film. When the spectra given in Figure S12 are examined, the O atom content of the polymer film is 27.66%, while the O atom content of ON–CDs increases to 33.82%. In addition, unlike the polymer film, which does not contain N atoms, the N content of PF(ON–CDs) is 6.41%. The increase in the contents of the O and N atoms confirms that the polymer film contains ON–CDs.

For the application of the prepared and characterized PF(ON–CDs), the film cut into suitable sizes (0.5 × 2.0 cm) as shown in Scheme 3b was placed separately in PA solutions prepared at different concentrations (5.0 × 10^–6^–50.0 × 10^–6^ M). The surfaces of PF(ON–CDs), which were left for 15 min for the interaction of ON–CDs with PA, were dried to avoid errors in the analysis. The blue fluorescence intensity of PF(ON–CDs) placed under a UV lamp at 365 nm in a dark medium was analyzed with a smartphone-supported application (“Colorimeter” application for Android OS, using a Samsung Galaxy A51 smartphone).

Stability tests were carried out to verify that the fluorescence intensity of the prepared polymer films was due to the interaction with PA and that the polymeric film did not leach out the carbon dots from its structure into the aqueous solution. First, the fluorescence intensity of 2.0 mL of distilled water at a wavelength of 455 nm was recorded. Afterward, the prepared polymer film was placed in this water, and the fluorescence intensity of the aqueous solution at a wavelength of 455 nm was recorded every 5 min (for 30 min). When the graphs given in Figure S13a (fluorescence intensity at 455 nm) and S13b (recovery % at 455 nm) are examined, it is seen that the fluorescence intensity of the aqueous solution with 12 fluorescence intensity increased to 13 after 15 min in the presence of polymer film, and this value was 15 after 30 min. Based on these values, the recovery values obtained at 455 nm wavelength are between 0.08 and 0.14%, which may also arise from tolerable experimental errors. This confirms that the carbon dots in the polymer film structure remain stable without passing into the aqueous medium and that the fluorescence reduction is due to the interaction of PA with ON–CDs.

**Figure 7:**
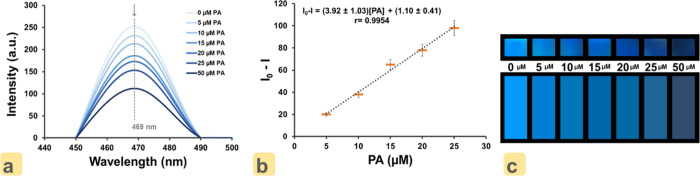
(a) Smartphone blue-emission intensity spectra of PF(ON–CDs) in the presence of varying concentrations of PA (5.0 × 10^–6^ 50 × 10^–6^ M, using colorimeter application). (b) Calibration plot of PF(ON–CDs) in the presence of PA (5.0 × 10^–6^–50 × 10^–6^ M). (c) Top photograph: The photographs of PF(ON–CDs) before and after interaction with picric acid (5.0 × 10^–6^–50 × 10^–6^ M) under a UV lamp at 365 nm. Bottom photograph: The color analysis photographs of PF(ON–CDs) before and after interaction with PA (5.0 × 10^–6^–50 × 10^–6^ M) using the “Colorimeter” application.

The blue fluorescence intensity of each PF(ON–CDs) placed under a UV lamp at 365 nm was analyzed with a smartphone-supported application (“Colorimeter” application for Android OS, using a Samsung Galaxy A51 smartphone). Each color measurement was performed three times with a smartphone placed 1.0 cm away from PF(ON–CDs) under the UV lamp. The graph given in Figure 7a was obtained with the spectra taken from the “Colorimeter” application. The fluorescence of PF(ON–CDs) film decreases with increasing PA concentration (5.0 × 10^–6^–50 × 10^–6^ M). Depending on this situation, the observed color intensity and the obtained color intensity spectrum decrease in the “Colorimeter” application. The color intensity decrement observed at 469 nm wavelength was plotted against the PA concentration. Figure 7b displays the curve of intensity difference against analyte concentration having a smooth linear range in the concentration range of PA from 5.0 × 10^–6^–25 × 10^–6^ M. Additionally, in Figure 7c, there are images of PF(ON–CDs) under a UV lamp (top photograph) and the colors of the fluorescence intensities of these PF(ON–CDs) under a UV lamp with “Colorimeter” application (bottom photograph). The equation for the linear variation of ON–CDs color intensity quenching (Δ*I*) with PA micromolar concentration (*C*_PA_) is given as
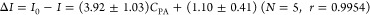
where *I*_0_ and *I* denote the fluorescence color intensities at 469 nm in the absence and presence of PA, respectively. The PA LOD was calculated to be 1.0 × 10^–6^ M (1.0 μM), and the CV were 1.88% (intra-assay) and 2.74% (inter-assay). The validation parameters regarding the analytical performance of ON–CDs for the determination of PA are briefed in Table 1.

## Conclusions

In this study, high QY (49.7%) ON–CDs as nanoprobes with superior properties were manufactured by simple refluxing with the use of CA, Glu, and EDA as precursors. The structure of ON–CDs, which has good aqueous solubility owing to the hydroxyl and amine groups on its surface, has been elucidated with different instrumental techniques, such as STEM, HRTEM, IR, Raman, XPS, and DLS. The ON–CDs with a mean particle size of 3.0 nm show high photostability and a bright blue fluorescence, the intensity of which does not change up to 50 days. The LOD value for PA determination based on emission measurements is 12.5 × 10^–12^ M (12 pM), which is lower than the values reported in most fluorometric assays found in the literature. In the determination of PA based on absorbance measurement, the LOD value is 9.0 × 10^–10^ M (0.9 nM). The LOD obtained by both methods is below the established permissible limits for phenolic compounds, which were set as 0.001 mg L^–1^ by the World Health Organization in drinking water and as 0.1 mg L^–1^ by the United States Environmental Protection Agency in wastewater. The effects of various cations, common anions, and camouflage materials were shown not to be significant in the determination of PA; i.e., PA could be recovered from admixture solutions at percentages ranging from 95.2 to 102.5%. For method validation, PA extracted from soil samples contaminated with PA was analyzed using both the proposed method and the LC–MS/MS reference method. For field applications, a PVA-based polymer composite film PF(ON–CDs) was prepared and used for the determination of PA in an aqueous medium. As a result of smartphone-assisted measurements, PF(ON–CDs) was able to determine PA at the micromolar level. PF(ON–CDs) is an important support material that makes ON–CDs functional and allows ON–CDs to determine PA not only in the laboratory but also in field applications. ON–CDs have the potential to play an important role in analytical nanoscience and nanotechnology for on-site and in-field use, due to their favorable optical characteristics, low cost, and high selectivity/sensitivity to the target analyte in complex environments.

## Supporting Information Available

Abbreviations; preparation of solutions; procedure of the LC–MS/MS method for validation of PA detection; optimization of experimental conditions; 3D model of STEM images with 30 nm zoom; fluorescence spectra and intensities of ON–CDs prepared at different temperatures and at different reaction times; fluorescence spectra and histogram of ON–CDs obtained at different excitation wavelengths, and ON–CDs prepared in different solvents and solvent mixtures; fluorescence intensity values of ON–CDs and ON–CDs/PA obtained at different buffer solutions and pH values; the relationship between *I*_0_–*I* of ON–CDs and increasing concentrations of PA in the solvent mixture medium and aqueous medium; fluorescence spectra and histogram of ON–CDs in the presence of various explosives; fluorescence spectra and histogram of ON–CDs without PA and with PA in the presence of different metal cations, anions, and camouflage materials; digital photographs of PVA-based polymer film and PF(ON–CDs) with different conditions under daylight and a UV lamp; XPS survey spectra of PVA-based polymer film and PF(ON–CDs); stability of PF(ON–CDs) in an aqueous medium; parameters of the quantum yield (QY) calculation of ON–CDs; comparison of the analytical performance parameters of fluorescent probes developed for PA detection; recovery values of PA in possible interference, such as metal cations, anions, and camouflage materials; molecular formulas and molecular masses of explosives; references (PDF)

## Abbreviations

ON–CDs: oxygen- and nitrogen-doped carbon dots
PF(ON–CDs): ON–CDs-based polymer PVA film
PA: picric acid (2,4,6-trinitrophenol)
TNT: 2,4,6-trinitrotoluene
HMX: 1,3,5,7-tetranitro-1,3,5,7-tetraazacyclooctane
RDX: 1,3,5-trinitroperhydro–1,3,5-triazine
PETN: pentaerythritol tetranitrate
NTO: 3-nitro-1,2,4-triazole-5-one
CA: citric acid
Glu: d-glucose
EDA: ethylenediamine
EtOH: ethyl alcohol
MeOH: methanol
DMSO: dimethyl sulfoxide
ACN: acetonitrile
HEPES: 2-[4-(2-hydroxyethyl)piperazine-1-yl]ethanesulfonic acid
Tris: 2-amino-2-(hydroxymethyl)-1,3-propanediol
H_2_O_2_: hydrogen peroxide
PBS: phosphate buffer solution
NH_4_Ac: ammonium acetate
NaH_2_PO_4_: sodium dihydrogen phosphate
Na_2_HPO_4_: disodium hydrogen phosphate
NaCH_3_COO: sodium acetate
CH_3_COOH: acetic acid
UPLC: ultraperformance liquid chromatography
MS: mass-spectroscopy
AFM: atomic force microscopy
STEM: scanning transmission electron microscopy
HRTEM: high-resolution transmission electron microscopy
