# Supplementary material for: Heteroatom-Doped Carbon Quantum Dots and Polymer Composite as Dual-Mode Nanoprobe for Fluorometric and Colorimetric Determination of Picric Acid
Source: ACS Appl Mater Interfaces. 2023 Aug 23;15(35):42066–79. doi: 10.1021/acsami.3c07938 (PMC10485801; doi:10.1021/acsami.3c07938)
Supplement: Supplementary file 1 — am3c07938_si_001.pdf [file am3c07938_si_001.pdf]

SUPPORTING INFORMATION

ACS APPLIED MATERIALS & INTERFACES

Heteroatom-Doped Carbon Quantum Dots and Polymer Composite as Dual-Mode Nanoprobe for Fluorometric and Colorimetric Determination of Picric Acid

Ömer Kaan Koç[Ψ,♀], Ayşem Üzer[♀,*], and Reşat Apak[♀,Φ,**]

[Ψ] Institute of Graduate Studies, Istanbul University-Cerrahpaşa, 34320 Avcilar, Istanbul, Turkey.

[♀] Department of Chemistry, Faculty of Engineering, Istanbul University-Cerrahpaşa, 34320 Avcilar, Istanbul, Turkey.

[Φ] Turkish Academy of Sciences (TUBA), Bayraktar Neighborhood, Vedat Dalokay St. No: 112, Çankaya, 06690 Ankara, Turkey.

*Corresponding Author; Prof. Dr. Ayşem Arda (E-mail: auzer@iuc.edu.tr)
**Co-corresponding Author; Prof. Dr. Reşat Apak (E-mail: rapak@istanbul.edu.tr)

Supporting Information Contains

Abbreviations; preparation of solutions; procedure of LC–MS/MS method for validation of PA detection; optimization of experimental conditions; 3D model of STEM images with 30 nm zoom; fluorescence spectra and intensities of ON–CDs prepared at different temperatures and at different reaction times; fluorescence spectra and histogram of ON–CDs obtained at different excitation wavelengths, and ON–CDs prepared in different solvents and solvent mixtures; fluorescence intensity values of ON–CDs and ON–CDs/PA obtained at different buffer solutions and pH values; the relationship between I_0-I of ON–CDs and increasing concentrations of PA in solvent mixture medium and aqueous medium; fluorescence spectra and histogram of ON-CDs in the presence of various explosives; fluorescence spectra and histogram of ON-CDs without PA and with PA in the presence of different metal cations, anions, and camouflage materials; digital photographs of PVA-based polymer film and PF(ON-CDs) with different conditions under daylight and UV-lamp; XPS survey spectra of PVA-based polymer film and PF(ON-CD s); stability of PF(ON-CDs) in aqueous-medium; parameters of the quantum yield (QY) calculation of ON-CDs; comparison of the analytical performance parameters of fluorescent probes developed for PA detection; recovery values of PA in admixtures with possible interferents such as metal ions, anions, and camouflage materials; molecular formulas and molecular masses of explosives; references.

Abbreviations

ON–CDs, oxygen and nitrogen-doped carbon dots; *PF(ON–CDs)*, ON–CDs -based polymer PVA film; *PA*, picric acid (2,4,6-trinitrophenol); *TNT*, 2,4,6-trinitrotoluene; *HMX*, 1,3,5,7–tetranitro–1,3,5,7–tetraazacyclooctane; *RDX*, 1,3,5–trinitroperhydro–1,3,5–triazine; *PETN*, pentaerythritol tetranitrate; *NTO*, 3-nitro-1,2,4-triazole-5-one; *CA*, citric acid; *Glu*, D-glucose; *EDA*, ethylenediamine; *EtOH*, ethyl alcohol; *MeOH*, methanol; *DMSO*, dimethyl sulfoxide; *ACN*, acetonitrile; *HEPES*, 2–[4–(2–hydroxyethyl)piperazine–1–yl]ethane sulfonic acid; *Tris*, 2–amino–2–(hydroxymethyl)–1,3–propanediol, H_2O_2, hydrogen peroxide; *PBS*, phosphate buffer solution, NH_4Ac, ammonium acetate; NaH_2PO_4, sodium dihydrogen phosphate; Na_2HPO_4, disodium hydrogen phosphate; $NaCH_3COO$, sodium acetate; CH_3COOH, acetic acid; *UPLC*, ultra-performance liquid chromatography; *MS*, mass-spectroscopy; *AFM*, atomic force microscopy; *STEM*, scanning transmission electron microscopy; *HRTEM*, high-resolution transmission electron microscopy.

Preparation of Solutions

The working solution of ON–CDs (0.01 g mL^{-1}) was prepared in ultrapure-water and stored at +4 °C for further experiments. HEPES–Tris (at pH 4.0–9.0) buffer solutions were prepared from 1.0×10^{-2} M HEPES and 1.0×10^{-2} M Tris. NH_4Ac (at pH 7.0) buffer solution was prepared from 1.0×10^{-2} M NH_4Ac. PBS (at pH 7.0) solution was prepared from 1.0×10^{-2} M NaH_2PO_4 and 1.0×10^{-2} M Na_2HPO_4. Acetate (at pH 7.0) buffer solution was prepared from 1.0×10^{-2} M $NaCH_3COO$ and 1.0×10^{-2} M CH_3COOH. The PA stock solution at 2.0×10^{-7} M was prepared in EtOH and stored at +4 °C. TNT, tetryl, RDX, Comp B (RDX), HMX, and NTO stock solutions at 2.0×10^{-7} M (for selectivity) and 4.0×10^{-6} M (for explosive mixture) were prepared in EtOH and stored at +4 °C. H_2O_2 stock solution at 2.0×10^{-7} M was prepared in ultrapure-water and stored at +4 °C. PETN stock solution at 2.0×10^{-7} M (for selectivity) and at 4.0×10^{-6} M (for explosive mixture) were prepared in EtOH:Acetone (1:1, *v*/*v*) and stored at +4 °C. The stock solutions of possible interferents such as metal cations (Cd^{2+}, Fe^{2+}, Mg^{2+}, Cu^{2+}, Zn^{2+}, Mn^{2+}, and Ca^{2+}) and anions (Cl^-, NO_3^-, NO_2^-, SO_4^{2-}, and CO_3^{2-}) were prepared separately at 2.0×10^{-5} M in ultrapure-water. The solutions of camouflage materials (detergent, glucose, aspartame, paracetamol, and acetylsalicylic acid) at 2.0×10^{-5} M as possible interferents were prepared in ultrapure-water.

Procedure of LC–MS/MS Method for Validation of PA Detection

The working solutions of PA at 0.44×10^{-6} M, 0.87×10^{-6} M, 1.75×10^{-6} M, 3.5×10^{-6} M, and 4.4×10^{-6} M were prepared from the corresponding stock solutions at 450.0×10^{-6} M in acetone. LC–MS analysis was performed on a UPLC–MS/MS equipment (Shimadzu, 20A) employing an injection volume of 15.0 μL. LC was equipped with a Rectek Ultra-AQ column (100×2.1 mm, 3 μm, C18). The column temperature was 40 °C; the injector temperature 4 °C, and the column flow rate 1.0 mL min^{-1}. Two different ammonium acetate solutions, each at 5 mM concentration, in ultrapure-water (mobile phase A) and in pure methanol (mobile phase B), were used as mobile phases A and B, respectively, at a flow rate of 0.3 mL min^{-1}. LC–MS/MS analysis was carried out using the negative ion mode electrospray ionization method, and the ionization voltage was 3.5 kV. The product ion and precursor ion were 240.7 m/z for PA (collision energy: 15.0 V).

Optimization of Experimental Conditions

Oxygen- and nitrogen-doped ON–CDs were prepared by a one-step reflux method using CA, Glu, and EDA as molecular precursors containing abundant hydroxyl (-OH) and amino ($-NH_2$) groups, respectively. The molecular precursors were put into the high-temperature reaction flask through a condensation polymerization step forming a polymer-like graphite sheet which was further carbonized to form ON–CDs. The hydroxyl- and amino-groups functionalized on this graphite layer surface are responsible for the fluorescence of ON–CDs. Extensive research has shown that the fluorescent properties of ON–CDs are strongly influenced by the synthesis technique. Therefore, various reaction conditions such as reaction temperature and time were investigated to optimize the fluorescent performance of ON–CDs. As shown in **Figure S2a and S2b**, the fluorescence intensity of the prepared ON–CDs first increased (160 °C to 180 °C) and then decreased (180 °C to 190 °C) with increasing reaction temperature. Similarly, as shown in **Figure S3a and S3b**, the fluorescence intensity of ON–CDs first increased (2 h to 3 h) and then decreased again (3 h to 5 h) with increasing reaction time. With increasing temperature, the reaction is more complete and results in an increased yield of ON–CDs **[1]**. In addition, bright fluorescent ON–CDs can be obtained with increasing reaction time. However, if the reaction temperature and time continue to increase, the formation rate and the particle size of the carbon cores will increase, which will reduce the fluorescence intensity of the

ON–CDs. Considering not only the optimal fluorescence property of the ON–CDs **[2]**, but also cost and energy savings, the final reaction temperature and time were determined as 180 °C and 3 h, respectively. Under these determined conditions, ON–CDs were prepared with a quantum yield of 49.7%.

Optimal wavelength and solvent system were investigated for ON–CDs prepared under optimized synthesis conditions. Emission spectra of ON–CDs excited at different wavelengths (310 nm to 420 nm) were recorded as shown in **Figure S4a**. When the recorded spectra were examined, the emission band with the highest fluorescence intensity was recorded at 355 nm excitation wavelength (**Figure S4b**). Also, in experimental studies, it is necessary to know the behavior of ON–CDs in different organic solvents (ethanol, methanol, dimethyl sulfoxide, and acetone) and mixtures with H_2O (1:1, *v*/*v*). When the spectra in **Figure S5a** and the fluorescence intensity histogram in **Figure S5b** are examined, it is seen that the best fluorescence intensity of ON–CDs is obtained in H_2O. However, since ON–CDs are used in the determination of explosives, the use of organic solvents is extremely important for the solubility of explosives. For this reason, EtOH was chosen as a suitable solvent, which was prepared as a mixture with water, and the EtOH-H_2O (1:1, *v*/*v*) solvent mixture was preferred throughout the experimental studies.

Another parameter that is extremely important for ON–CDs is the pH of the medium, because the characteristic structure of ON–CDs and the way it responds in the presence of the target analyte may vary according to the ambient pH. For this reason, emission spectra of ON–CDs at 455 nm wavelength were recorded in the absence and presence of PA with HEPES-Tris buffer solution within a pH range of 4 to 9. As seen in the fluorescence intensity histogram given in **Figure S6a**, ON–CDs maintains fluorescence stability in the pH range of 4 to 9. However, in the presence of PA, the best signal was recorded at pH 7. After determining pH 7 as the most suitable working medium for ON–CDs, experiments were carried out with different buffers (HEPES-Tris, NH_4Ac, PBS, and acetic acid-acetate) that would fix the working medium to pH 7. Fluorescence intensities were recorded in pH 7 medium with different buffer systems of ON–CDs in the absence (**Figure S6b**) and presence (**Figure S6c**) of PA. As a result of the results obtained in the histograms, it was determined that the best analytical signals were obtained with HEPES-Tris (pH 7) buffer, which was used throughout the experimental studies.

Supplementary Figures

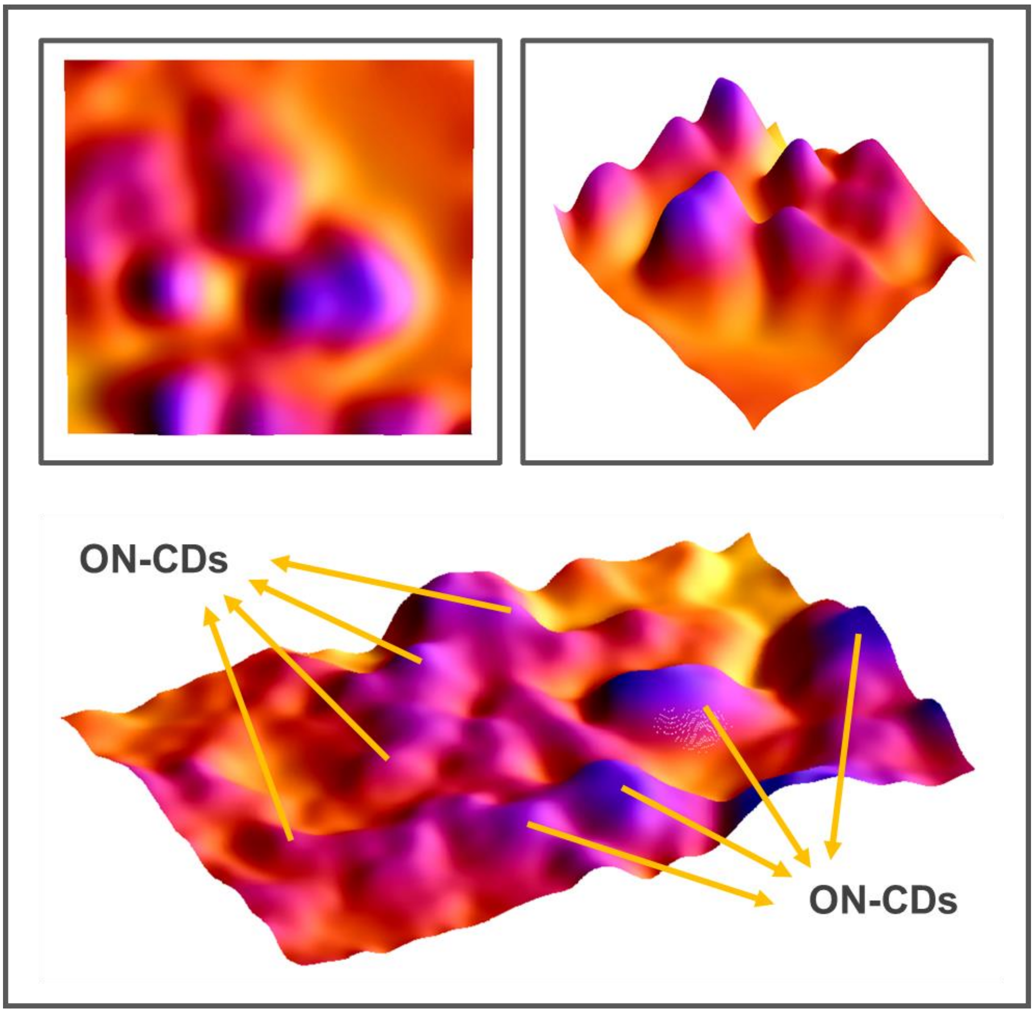

Figure S1.

AFM Example of virtual Thermal LUT of HRTEM image (Figure 2c) with 20 nm zoom of the ON–CDs. (The 3D model was performed using ImageJ software following this step: Open HR-TEM image; 3D surface plot; Thermal LUT.)

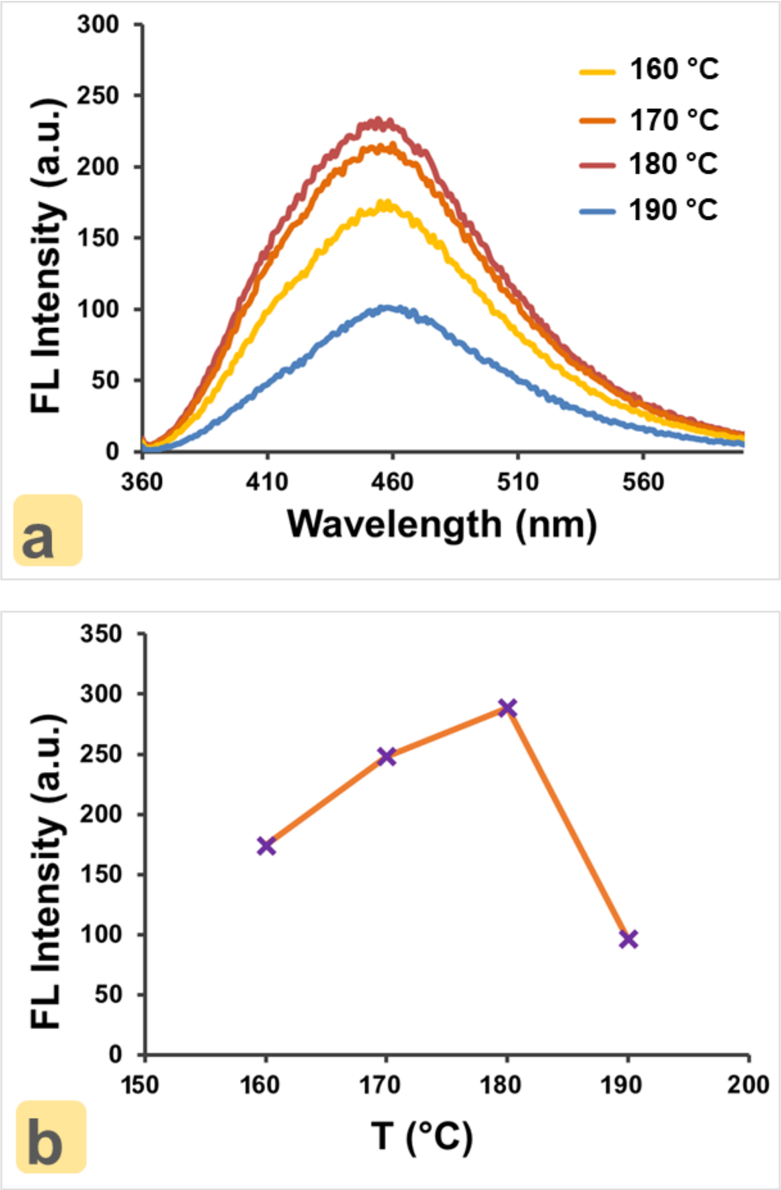

Figure S2.

Fluorescence **a)** spectra and **b)** intensity of the ON–CDs with different temperatures at the excitation and emission wavelengths of 355 nm and 455 nm, respectively.

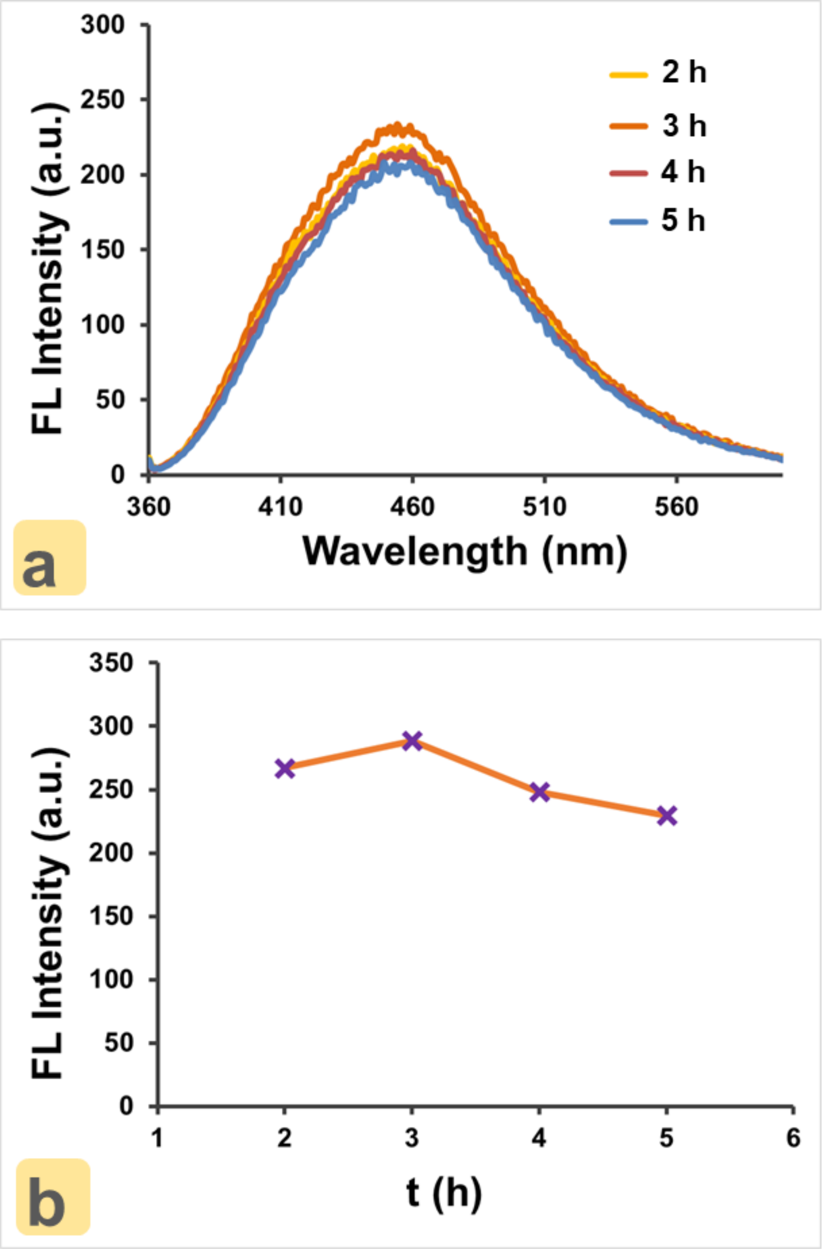

Figure S3.

Fluorescence **a)** spectra and **b)** intensity of the ON–CDs with different reaction times at the excitation and emission wavelengths of 355 nm and 455 nm, respectively.

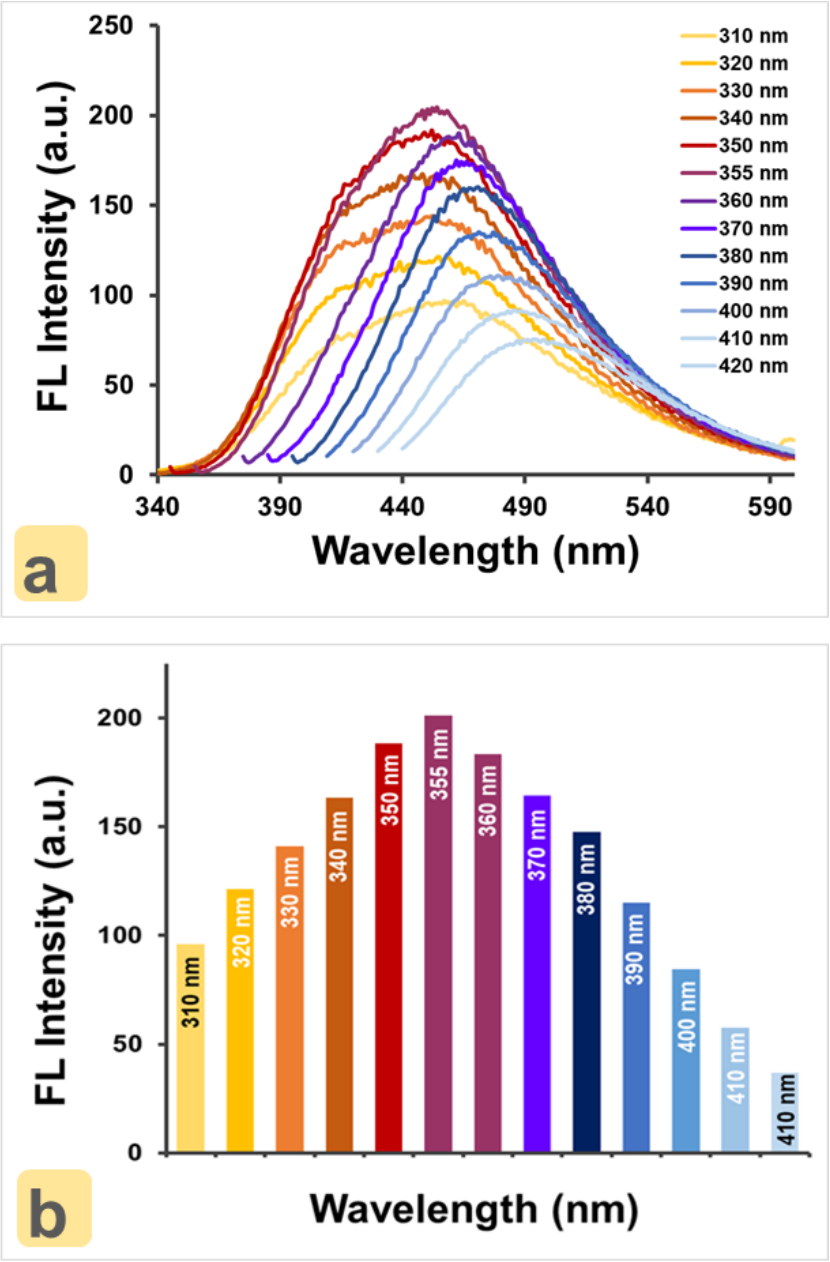

Figure S4.

Fluorescence **a)** spectra and **b)** intensities of ON–CDs at different excitation wavelengths.

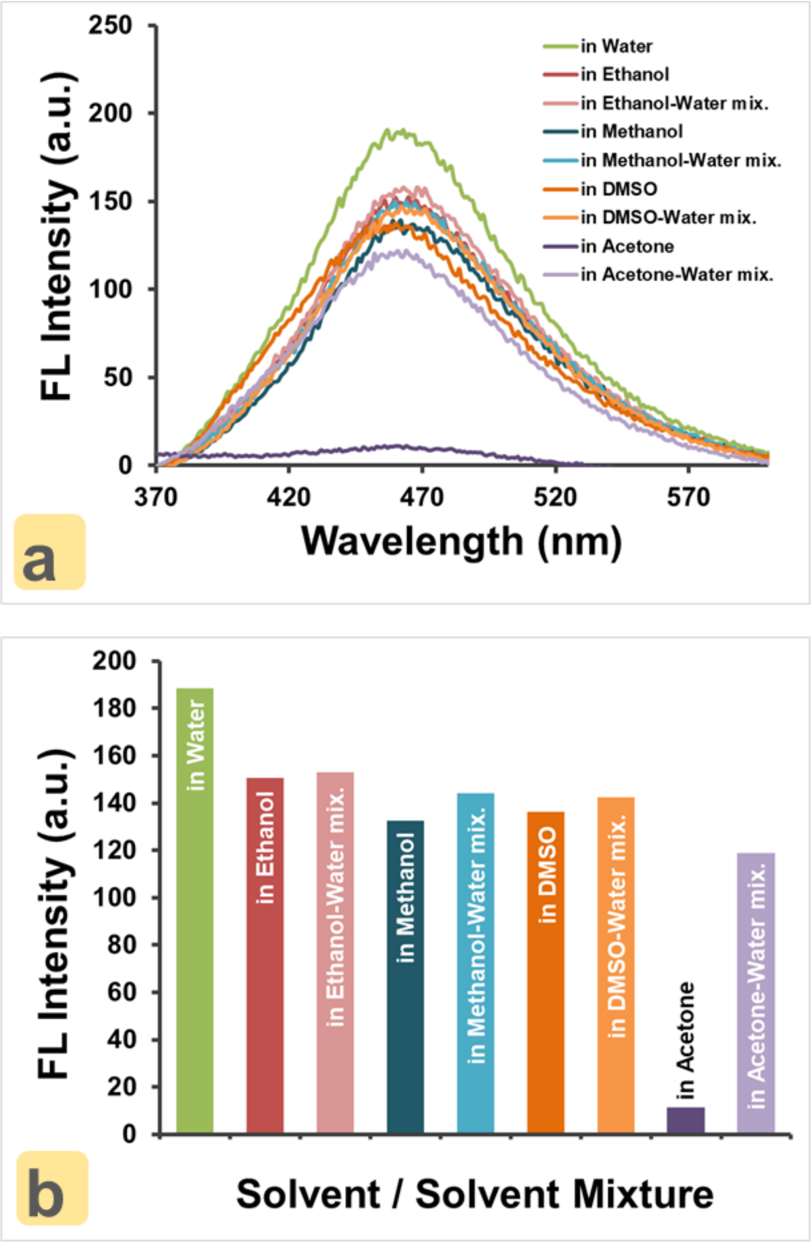

Figure S5.

Fluorescence **a)** spectra and **b)** intensities of ON–CDs in different solvents and solvent mixtures (The volume ratio of EtOH-H_2O, MeOH-H_2O, DMSO-H_2O, and acetone-H_2O solvent mixtures is 1:1).

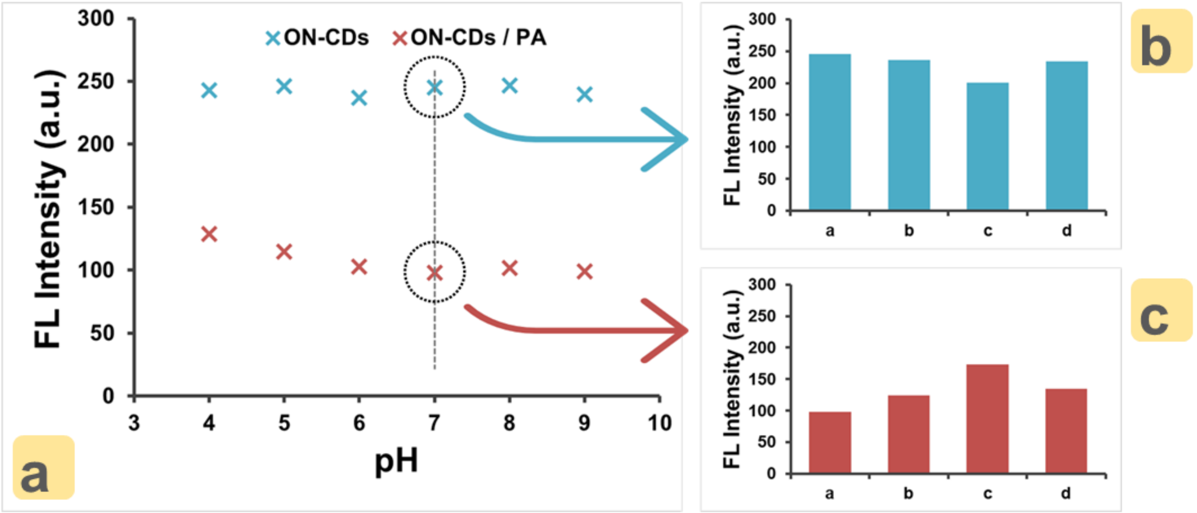

Figure S6.

a) Fluorescence intensities of the ON–CDs (blue signs) alone and ON–CDs (red signs) in the presence of PA (10×10^{-9} M) at different pH from 4 to 10 (HEPES-Tris buffer, 1.0×10^{-2} M). Fluorescence intensity histograms of **b)** ON–CDs (blue signs) and **c)** ON–CDs (red signs) in the presence of PA (10×10^{-9} M) in different buffer solutions (a: HEPES-Tris, b: NH_4Ac, c: PBS, and d: acetate buffer at pH 7.0).

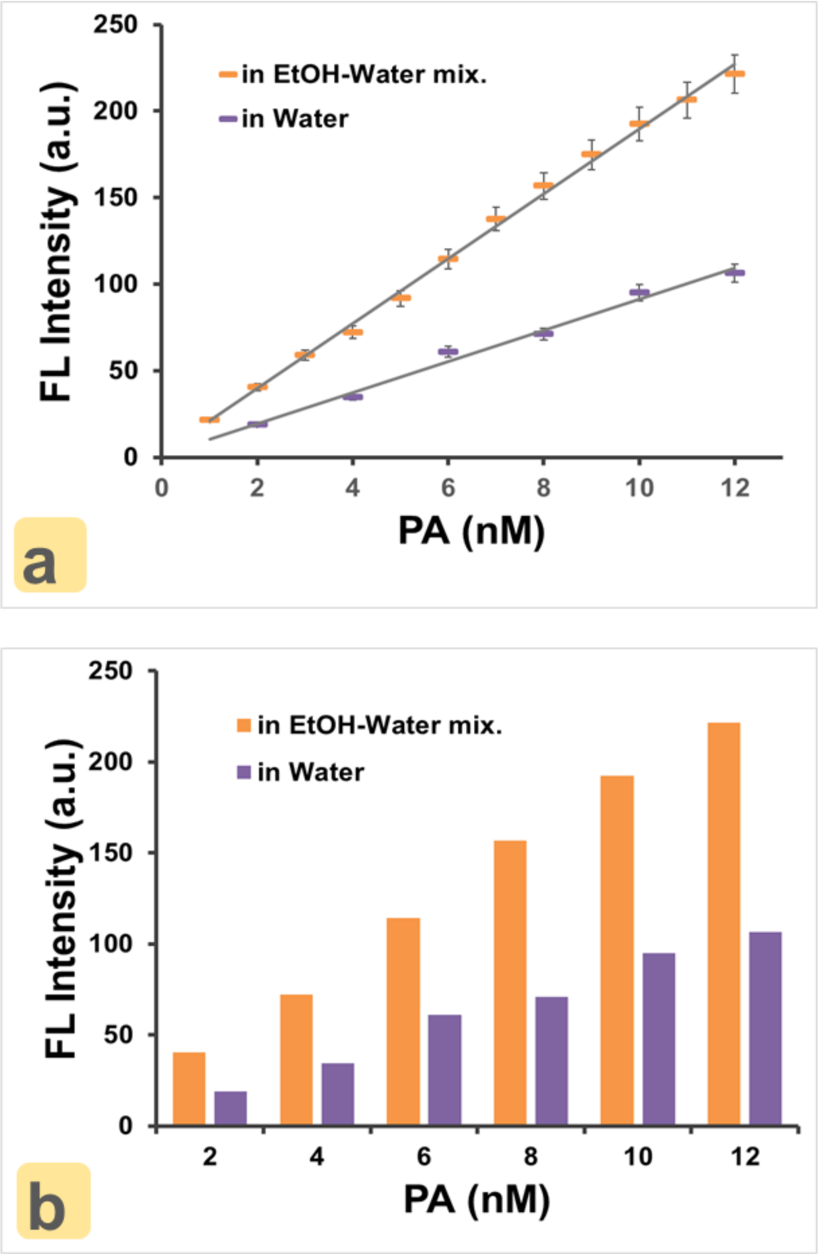

Figure S7.

The relationship between I_0-I (ΔI) and increasing concentrations of PA in EtOH-H_2O (1:1, v/v) solvent mixture medium (orange line for S7a and orange bars for S7b) and H_2O medium (purple line for S7a and purple bars for S7b), where I_0 and I are **a)** the fluorescence intensities and **b)** intensity histograms of ON–CDs in the absence and presence of PA, respectively.

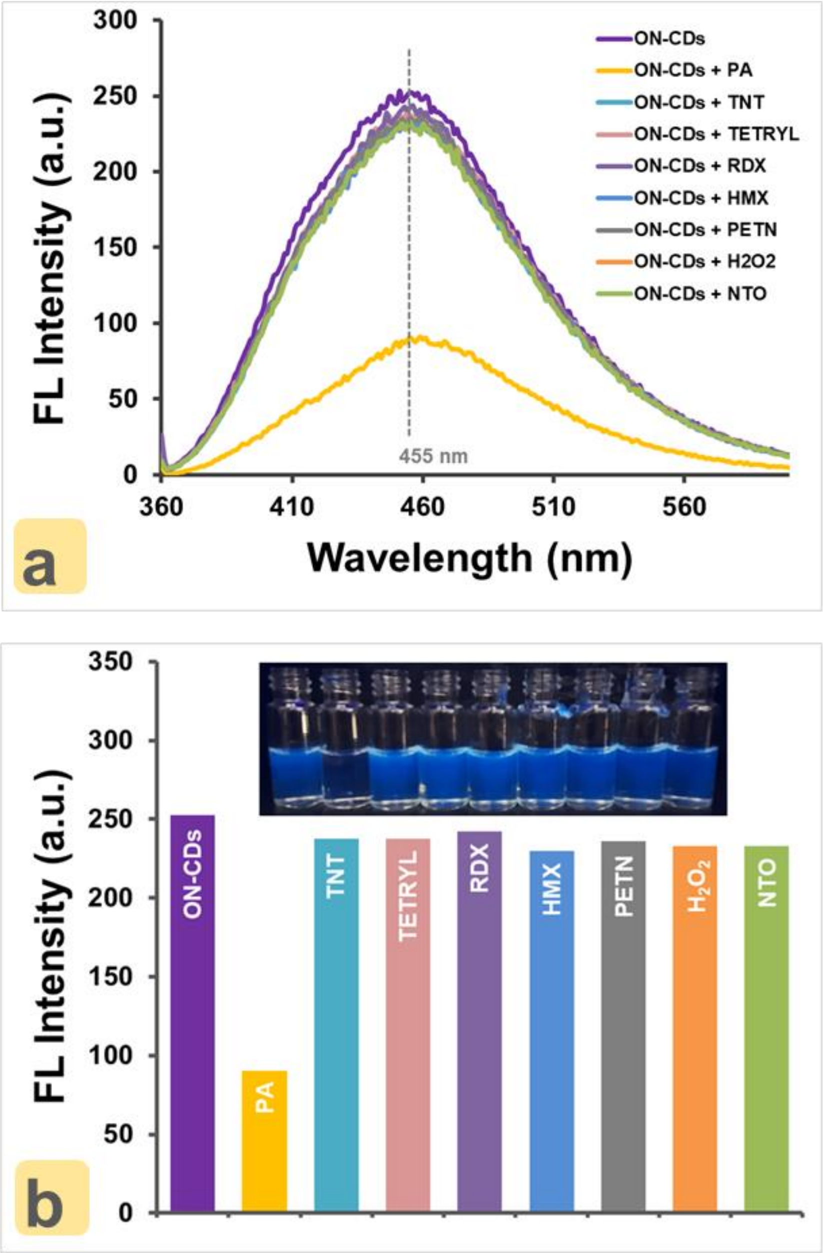

Figure S8.

Fluorescence **a)** spectra and **b)** intensity histogram (inset: photographs of ON–CDs by adding different explosives under UV-lamp at 365 nm) of ON–CDs in the presence of various explosives and hydrolyzates such as PA, TNT, tetryl, RDX, HMX, PETN, H_2O_2, and NTO.

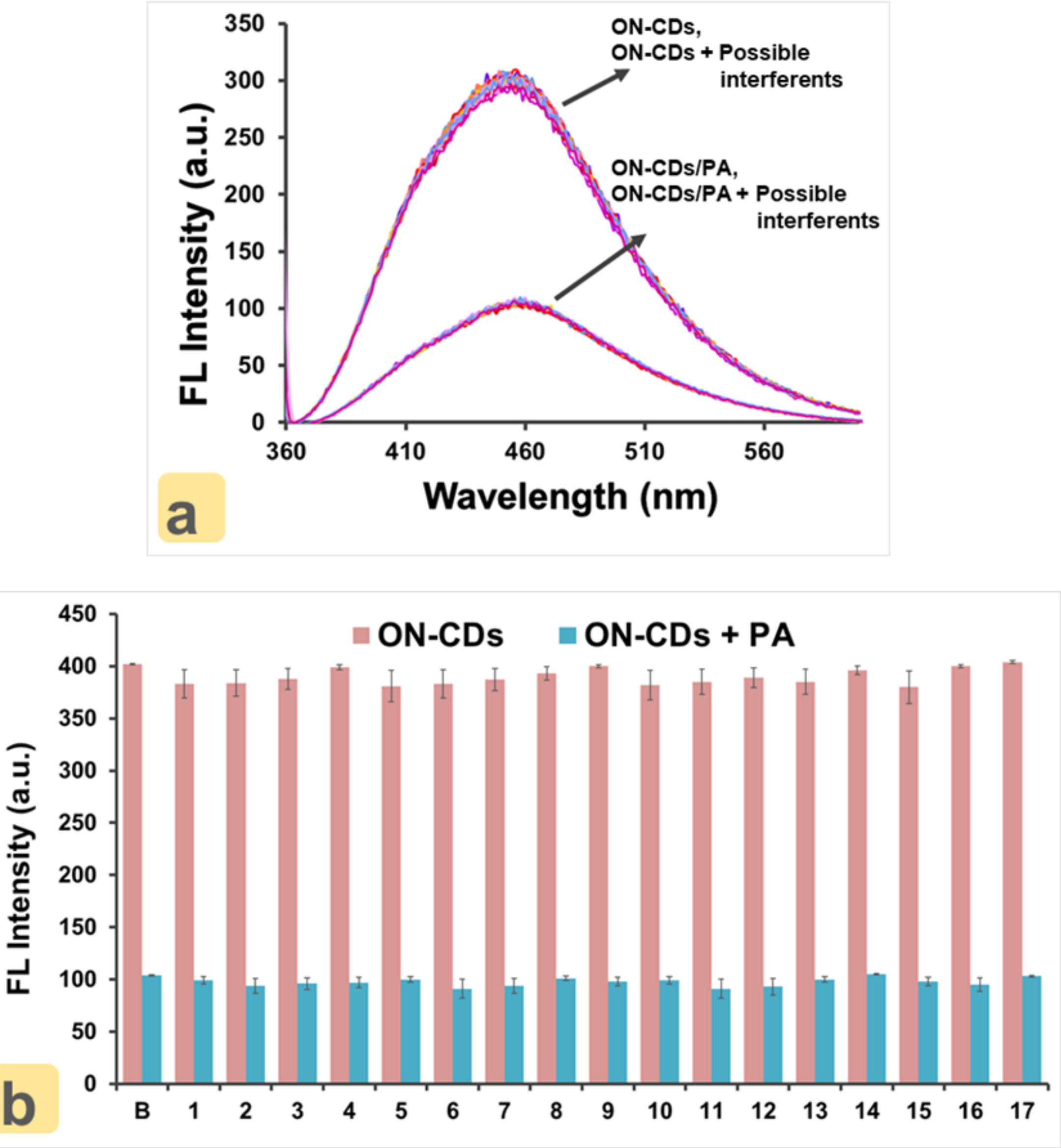

Figure S9.

Fluorescence **a)** spectra and **b)** intensity of ON–CDs without PA (pink bars) and with PA (blue bars) in the presence of different metal cations (at 100-fold, number 1 to 7), anions (at 100-fold, number 8 to 12), and camouflage materials (at 25-fold, number 13 to 17) {B: blank, 1: Cd^{2+}, 2: Fe^{2+}, 3: Mg^{2+}, 4: Cu^{2+}, 5: Zn^{2+}, 6: Mn^{2+}, 7: Ca^{2+}, 8: Cl^-, 9: NO_3^-, 10: NO_2^-, 11: SO_4^{2-} 12: CO_3^{2-}, 13: glucose, 14: detergent, 15: aspartame, 16: paracetamol, and 17: acetylsalicylic acid).

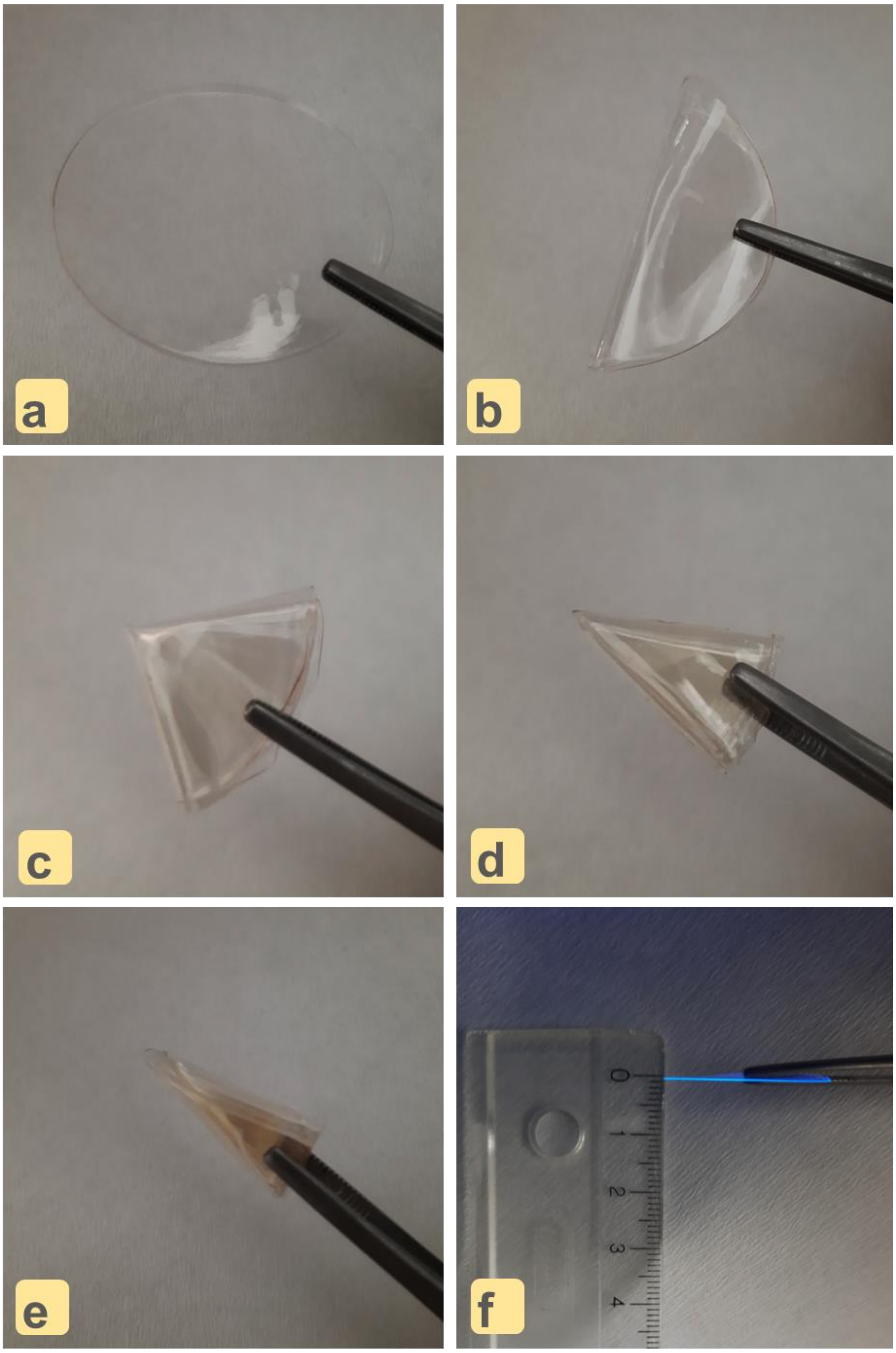

Figure S10.

Digital photographs of PF(ON–CDs) with different conditions under **a)**-**e)** daylight (S8a to S8e) and **f)** UV-lamp at 365 nm.

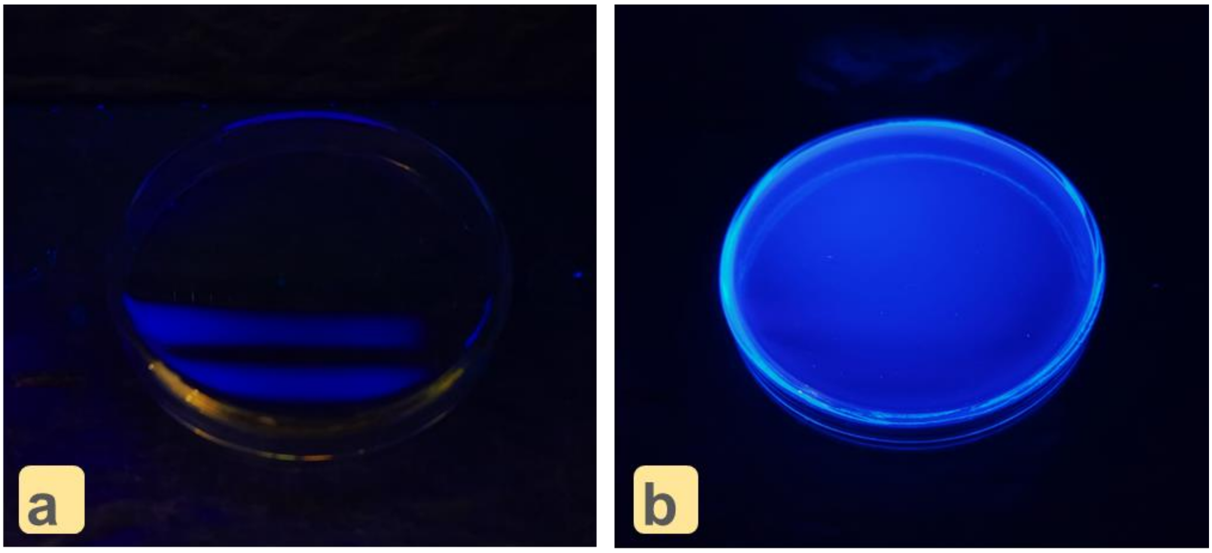

Figure S11.

Digital photograph of **a)** PVA-based polymer film and **b)** PF(ON–CDs) under UV-lamp at 365 nm.

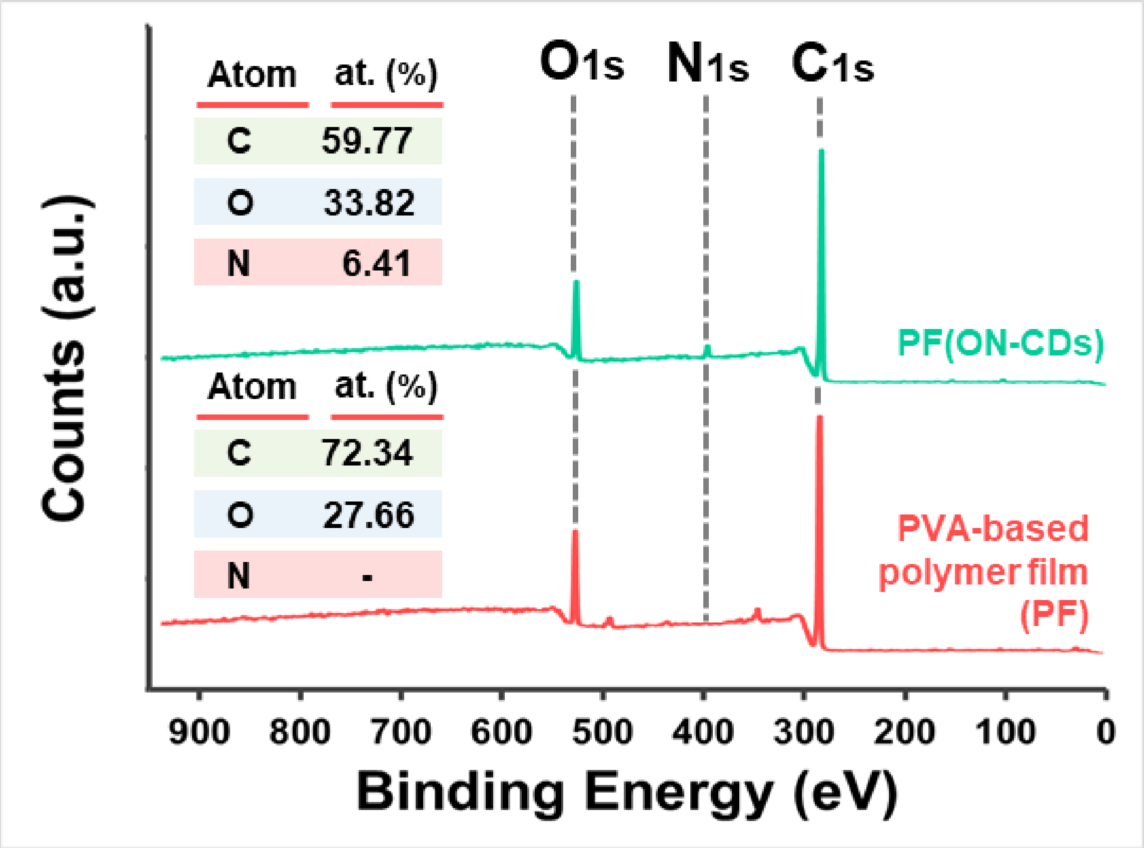

Figure S12.

XPS survey spectra of PVA-based polymer film (red line) and PF(ON–CDs) (green line).

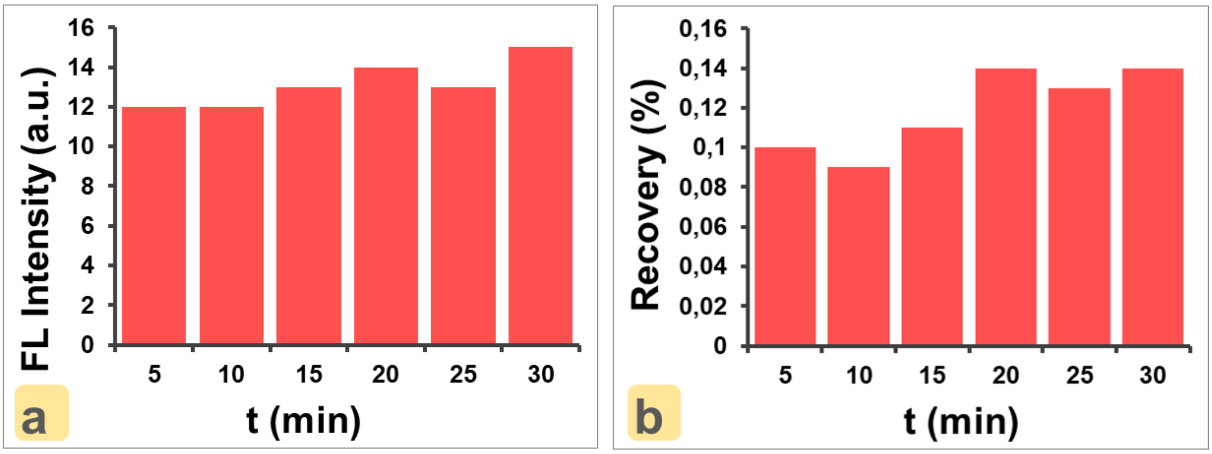

Figure S13.

a) Fluorescence intensities and **b)** recovery (%) values at 455 nm wavelength recorded at different times (5 min to 30 min) of the aqueous-solution containing the PF(ON–CDs).

Supplementary Tables

Table S1

Quantum yield (QY) of the fluorescent nanoprobe ON–CDs.

**Sample**	**Integrated emission intensity (*I*)**	**Abs. at 355 nm (*A*)**	**Refractive index of solvent (*n*)**	**Quantum yield at 355 nm (*Q*)**
Anthracene	1012.8	0.093	1.36	27.0 % (known)
ON–CDs	1889.2	0.090	1.33	49.7 %

Table S2

Comparison of properties of different fluorescent probes.

Fluorescence Probes	Carbon Source	Hetero-atom Source	Synthesis Method	Particle Size (nm)	Quantum Yield (%)	Analyte	Analysis Method	Ref.
CDs	Citric Acid	Acrylamide	Hydrothermal	8.6	55.4	Fe^{3+}, Cr^{6+}, and Ascorbic Acid	Fluorometric	**[3]**
N-CDs	Citric Acid	*o*-Phenylenediamine	Solar Irradiation	4.31	54.0	Hg^{2+}	Fluorometric	**[4]**
CDs	Citric Acid	Ethylenediamine	Hydrothermal	5.0	-	Hemoglobin	Fluorometric	**[5]**
S, N co-doped C-dots	Citric Acid	Thiourea	Hydrothermal	4.0	73.1	Uric Acid	Fluorometric	**[6]**
CDs	Citric Acid	Polyacrylamide	Hydrothermal	4.1	12.6	Picric Acid	Fluorometric	**[7]**
ON-CDs	Citric Acid	Ethylenediamine and D-Glucose	Reflux	3.0	49.7	Picric Acid	Fluorometric and Colorimetric	**This work**

Table S2 shows probes based on carbon dots using citric acid as the carbon-source with the use of different molecular precursors as heteroatom sources. The synthesized carbon dots are mostly N-doped and contain a high amount of amine groups ($-NH_2$) on the surface. On the other hand, ON-CDs have a high amount of hydroxyl groups (-OH) in addition to the amine group ($-NH_2$) on its surface. The methods of synthesis mostly include thermal methods (i.e., hydrothermal). Unlike these synthesis methods, ON-CDs were synthesized by the reflux method and may be an alternative to the literature. Looking at the particle sizes, the average smallest particle size belongs to ON-CDs. Considering the reported quantum yields, ON-CDs have a very high quantum yield. Although there are carbon dots with higher quantum efficiency in the literature, ON-CDs has a simple synthesis method, more than one heteroatom doped on its surface, shows high photostability, and allows practical application by forming composite material with support material (polymer film). In addition, carbon dot-based probes are mostly used as fluorescent probes, but our selective ON-CDs can determine the analyte both

fluorometrically and colorimetrically, owing to their high hydroxyl group content and to the balance between surface hydroxyls and amines. To the best of our knowledge, this is the first time use of an oxygen-rich CDs-sensor owing to the use of glucose as a precursor **[8]**.

Table S3

Comparison of sensing performance of different fluorescent probes for PA detection.

Fluorescence Probes	Linear Range (μM)	Correlation Coefficient (R^2)	Detection Limit (nM)	References
Fe-doped ZnO NEs	5.0 – 60.0	–	2930	**[9]**
Amine-capped carbon dots	-	-	1000	**[10]**
Rhodamine-B based molecular probe	10.0 – 50.0	0.9874	820	**[11]**
Nitrogen doped graphene quantum dots	0.0 – 4.0	0.9934	420	**[12]**
Fluorescent amino-functionalized Zn(II)-MOF	0.0 – 25.0	0.9950	320	**[13]**
Polyimide covalent organic framework (COF)	0.5 – 10.0	0.9901	250	**[14]**
MoS_2 QDs	0.099 – 36.5	–	95	**[15]**
Pyridine based molecular probe	0.5 – 2.5	0.976	31.5	**[16]**
Cationic pyrene derivative molecular probe	0.0 – 0.4	0.9880	23.2	**[17]**
Carbon Dots	0.025 – 15.0<br>15.0 – 65.0	0.9998<br>0.9974	5.37	**[18]**
Ag nanoclusters	0.001 – 1.0	0.9962	0.1	**[19]**
Silicon nanodots (SiNDs)	8.0 – 50.0	0.9954	0.92	**[20]**
O– and N–doped carbon dots (ON–CDs)	0.001 – 0.011	0.9987	0.0125 (12.5 pM)	**This work**

ZnO: Zinc oxide, **NEs:** Nano-ellipsoids, **MOF:** Metal-organic framework, **MoS_2:** Molybdenum disulfide, **QDs:** Quantum dots, **Ag:** Silver, **μM:** Micro-molar, **nM:** Nano-molar, **pM:** Pico-molar, **–**: Represents the corresponding data is undetermined or unmentioned in the references.

Table S4

Recovery (%) of PA with the developed nanoprobe in the presence of metal cations (at 100-fold), anions (at 100-fold), and camouflage materials (at 25-fold).

**Metal Cations**		**Anions**		**Camouflage Materials**	
**Interferent**	**Recovery (%)**	**Interferent**	**Recovery (%)**	**Interferent**	**Recovery (%)**
Cd^{2+}	96.7	Cl^-	102.5	Glucose	98.1
Fe^{2+}	96.8	NO_3^-	100.3	Detergent	101.5
Mg^{2+}	97.5	NO_2^-	96.3	Aspartame	95.2
Cu^{2+}	100.5	SO_4^{2-}	97.0	Paracetamol	99.2
Zn^{2+}	95.3	CO_3^{2-}	96.8	Acetyl Salicy. Acid	100.3
Mn^{2+}	96.9				
Ca^{2+}	101.6				

Molecular Formulas and Molecular Masses

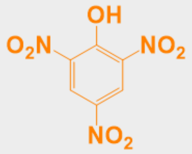

2,4,6-Trinitrophenol (TNP)
Picric Acid (PA)

229.10 g.mol^{-1}

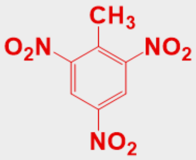

2,4,6-Trinitrotoluene (TNT)

227.13 g.mol^{-1}

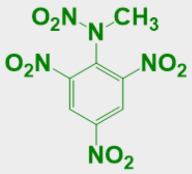

2,4,6–Trinitrophenylmethylnitramine (TETRYL)

287.15 g.mol^{-1}

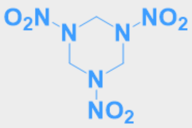

1,3,5–Trinitroperhydro–1,3,5–triazine (RDX)

222.12 g.mol^{-1}

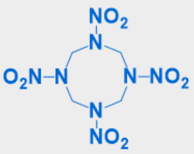

1,3,5,7–Tetranitro–1,3,5,7–tetraazacyclooctane (HMX)

296.15 g.mol^{-1}

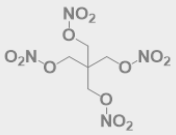

Pentaerythritol tetranitrate (PETN)

316.14 g.mol^{-1}

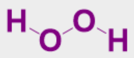

Hydrogen peroxide (H_2O_2)

34.01 g.mol^{-1}

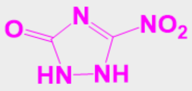

3-Nitro-1,2,4-triazole-5-one (NTO)

130.06 g.mol^{-1}

References

[1]

Zhu, S.; Shao, J.; Song, Y.; Zhao, X.; Du, J.; Wang, L.; Wang, H.; Zhang, K.; Zhang, J; Yang, B. Investigating the surface state of graphene quantum dots. *Nanoscale* 2015, 7, 7927-7933.

https://doi.org/10.1039/C5NR01178G

[2]

Fan, Y.Z.; Zhang, Y.; Li, N.; Liu, S.G.; Liu, T.; Li, N.B.; Luo, H.Q. A facile synthesis of water-soluble carbon dots as a label-free fluorescent probe for rapid, selective and sensitive detection of picric acid. *Sens. Actuators B Chem.* 2017, 240, 949-955.

https://doi.org/10.1016/j.snb.2016.09.063

[3]

Li, C.; Liu, W.; Sun, X.; Pan, W.; Wang, J. Multi sensing functions integrated into one carbon-dot based platform via different types of mechanisms. *Sens. Actuators B Chem.* 2017, 252, 544-553.

https://doi.org/10.1016/j.snb.2017.06.036

[4]

Lu, D.; Tang, Y.; Gao, J.; Chen, Y.; Wang, Q. Green anhydrous assembly of carbon dots via solar light irradiation and its multi-modal sensing performance. *Dyes Pigm.* 2019, 165, 287-293.

https://doi.org/10.1016/j.dyepig.2019.02.037

[5]

Barati, A.; Shamsipur, M.; Abdollahi, H. Hemoglobin detection using carbon dots as a fluorescence probe. *Biosens. Bioelectron.* 2015, 71, 470-475.

https://doi.org/10.1016/j.bios.2015.04.073

[6]

Wang, H.; Lu, Q.; Hou, Y.; Liu, Y.; Zhang, Y. High fluorescence S, N co-doped carbon dots as an ultra-sensitive fluorescent probe for the determination of uric acid. *Talanta* 2016, 155, 62-69.

https://doi.org/10.1016/j.talanta.2016.04.020

[7]

Wang, X.; Liu, Y.; Zhou, Q.; Sheng, X.; Sun, Y.; Zhou, B.; Zhao, J.; Guo, J. A reliable and facile fluorescent sensor from carbon dots for sensing 2,4,6-trinitrophenol based on inner filter effect. *Sci. Total Environ.* 2020, 720, 137680. https://doi.org/10.1016/j.scitotenv.2020.137680

[8]

Seven, E.S.; Cilingir, E.K.; Bartoli, M.; Zhou, Y.; Sampson, R.; Shi, Y.; Peng, Z.; Pandey, R.R.; Chusuei, C.C.; Tagliaferro, A.; Vanni, S.; Graham, R.M.; Seven, Y.B.; Leblanc, R.M. Hydrothermal vs microwave nanoarchitechtonics of carbon dots significantly affects the structure, physicochemical properties, and anti-cancer activity against a specific neuroblastoma cell line. *J. Colloid Interface Sci.* 2023, 630, 306-321. https://doi.org/10.1016/j.jcis.2022.10.010

[9]

Kumar, R.; Umar, A.; Rana, D.S.; Sharma, P.; Chauhan, M.S.; Chauhan, S. Fe-doped ZnO nanoellipsoids for enhanced photocatalytic and highly sensitive and selective picric acid sensor. *Mater. Res. Bull.* 2018, 102, 282-288. https://doi.org/10.1016/j.materresbull.2018.02.042

[10]

Niu, Q.; Gao, K.; Lin, Z.; Wu, W. Amine-capped carbon dots as a nanosensor for sensitive and selective detection of picric acid in aqueous solution *via* electrostatic interaction. *Anal. Methods* 2013, 5, 6228-6233. https://doi.org/10.1039/C3AY41275J

[11]

Zhang, Z.; Ju, P.; Guo, P.; Hou, X.; Hou, X.; Lv, H.; Wang, J.; Zhang, Y. A FRET-based fluorescent and colorimetric probe for the specific detection of picric acid. *RSC Adv.* 2018, 8, 31658-31665. https://doi.org/10.1039/C8RA05468A

[12]

Kaur, M.; Mehta, S.K.; Kansal, S.K. Nitrogen doped graphene quantum dots: Efficient fluorescent chemosensor for the selective and sensitive detection of 2,4,6-trinitrophenol.

Sens. Actuators B Chem. 2017, 245, 938-945.
https://doi.org/10.1016/j.snb.2017.02.026

[13]

Wiwasuku, T.; Boonmak, J.; Siriwong, K.; Ervithayasuporn, V.; Youngme, S. Highly sensitive and selective fluorescent sensor based on a multi-responsive ultrastable amino-functionalized Zn(II)-MOF for hazardous chemicals. *Sens. Actuators B Chem.* 2019, 284, 403-413.
https://doi.org/10.1016/j.snb.2018.12.094

[14]

Zhang, C.; Zhang, S.; Yan, Y.; Xia, F.; Huang, A.; Xian, Y. Highly Fluorescent Polyimide Covalent Organic Nanosheets as Sensing Probes for the Detection of 2,4,6-Trinitrophenol. *ACS Appl. Mater. Interfaces* 2017, 9, 15.
https://doi.org/10.1021/acsami.6b16423

[15]

Wang, Y.; Ni, Y. Molybdenum Disulfide Quantum Dots as a Photoluminescence Sensing Platform for 2,4,6-Trinitrophenol Detection. *Anal. Chem.* 2014, 86, 15, 7463–7470.
https://doi.org/10.1021/ac5012014

[16]

Ma, Y.; Zhang, Y.; Liu, X.; Zhang, Q.; Kong, L.; Tian, Y.; Li, G.; Zhang, X.; Yang, J. AIE-active luminogen for highly sensitive and selective detection of picric acid in water samples: Pyridyl as an effective recognition group. *Dyes Pigm.* 2019, 163, 1-8.
https://doi.org/10.1016/j.dyepig.2018.11.034

[17]

Liang, H.; Yao, Z.; Gea, W.; Qiaoc, Y.; Zhangc, L.; Cao, Z.; Wu, H.C. Selective and sensitive detection of picric acid based on a water-soluble fluorescent probe. *RSC Adv.*, 2016, 6, 38328-38331.
https://doi.org/10.1039/C6RA04080B

[18]

Wang, X.; Liu, Y.; Zhou, Q.; Sheng, X.; Sun, Y.; Zhou, B.; Zhao, J.; Guo, J. A reliable and facile fluorescent sensor from carbon dots for sensing 2,4,6-trinitrophenol based on inner

filter effect Sci. Total Environ. 2020, 720, 137680.
https://doi.org/10.1016/j.scitotenv.2020.137680

[19]

Zhang, J.R.; Yue, Y.Y.; Luo, H.Q.; Li, N.B. Supersensitive and selective detection of picric acid explosive by fluorescent Ag nanoclusters. *Analyst* 2016, 141, 1091-1097.
https://doi.org/10.1039/C5AN02251G

[20]

Qi, W.; He, H.; Fu, Y.; Zhao, M.; Qi, L.; Hu, L.; Liu, C.; Li, R. Water-dispersed fluorescent silicon nanodots as probes for fluorometric determination of picric acid via energy transfer. *Microchim Acta,* 2019, 186, 18.
https://doi.org/10.1007/s00604-018-3135-5
